# Organism-Adapted Specificity of the Allosteric Regulation of Pyruvate Kinase in Lactic Acid Bacteria

**DOI:** 10.1371/journal.pcbi.1003159

**Published:** 2013-07-25

**Authors:** Nadine Veith, Anna Feldman-Salit, Vlad Cojocaru, Stefan Henrich, Ursula Kummer, Rebecca C. Wade

**Affiliations:** 1 Molecular and Cellular Modelling Group, Heidelberg Institute for Theoretical Studies (HITS), Heidelberg, Germany; 2 Department of Modelling Biological Processes, Centre for Organismal Studies (COS)/BIOQUANT, Heidelberg University, Heidelberg, Germany; 3 Center for Modelling and Simulation in the Biosciences (BIOMS), Heidelberg, Germany; 4 Center for Molecular Biology (ZMBH), Heidelberg University, Heidelberg, Germany; University of Houston, United States of America

## Abstract

Pyruvate kinase (PYK) is a critical allosterically regulated enzyme that links glycolysis, the primary energy metabolism, to cellular metabolism. Lactic acid bacteria rely almost exclusively on glycolysis for their energy production under anaerobic conditions, which reinforces the key role of PYK in their metabolism. These organisms are closely related, but have adapted to a huge variety of native environments. They include food-fermenting organisms, important symbionts in the human gut, and antibiotic-resistant pathogens. In contrast to the rather conserved inhibition of PYK by inorganic phosphate, the activation of PYK shows high variability in the type of activating compound between different lactic acid bacteria. System-wide comparative studies of the metabolism of lactic acid bacteria are required to understand the reasons for the diversity of these closely related microorganisms. These require knowledge of the identities of the enzyme modifiers. Here, we predict potential allosteric activators of PYKs from three lactic acid bacteria which are adapted to different native environments. We used protein structure-based molecular modeling and enzyme kinetic modeling to predict and validate potential activators of PYK. Specifically, we compared the electrostatic potential and the binding of phosphate moieties at the allosteric binding sites, and predicted potential allosteric activators by docking. We then made a kinetic model of *Lactococcus lactis* PYK to relate the activator predictions to the intracellular sugar-phosphate conditions in lactic acid bacteria. This strategy enabled us to predict fructose 1,6-bisphosphate as the sole activator of the *Enterococcus faecalis* PYK, and to predict that the PYKs from *Streptococcus pyogenes* and *Lactobacillus plantarum* show weaker specificity for their allosteric activators, while still having fructose 1,6-bisphosphate play the main activator role *in vivo*. These differences in the specificity of allosteric activation may reflect adaptation to different environments with different concentrations of activating compounds. The combined computational approach employed can readily be applied to other enzymes.

## Author Summary

Some lactic acid bacteria are antibiotic resistant pathogens causing severe diseases whereas others are healthy probiotics used in the food industry. What makes an LAB a friend or a foe and how do they adapt to survive in such different environments? Here, we addressed this problem by focusing on the enzyme *pyruvate kinas*e, which plays a central role in the metabolism of lactic acid bacteria. This enzyme needs to react quickly to changes in the environment and, therefore, its activity is strictly regulated. In this study, we used computational techniques to predict the cellular substances, called allosteric activators that are responsible for the quick and effective activation of pyruvate kinase. We modeled the three dimensional structures of pyruvate kinases from different bacteria and computed interactions with possible activators. We simulated the dynamic behavior of the pyruvate kinase activity and, considering the cellular concentrations of metabolites in the different organisms, predicted the activators. We found that different lactic acid bacteria have different preferences for activators and that the level of activator specificity can be related to the environment in which the bacteria live.

## Introduction

Cellular metabolism comprises many different enzymatic reactions and pathways that need to work together in an orchestrated way. Organisms develop complex regulatory mechanisms in order to maintain the functional balance between individual pathways. The control mechanisms need to be flexible to allow the organism to react and adapt to perturbations such as changes in the environmental conditions. Therefore, different regulatory mechanisms have developed ranging from those at the metabolic or enzyme level to those at the genetic level. These operate on different timescales. A first, rapid level of response to environmental changes can be achieved by targeting the enzymatic reactions, thereby affecting enzyme activity directly [1].

Enzymatic reactions follow kinetic and thermodynamic rules. The thermodynamics imply among others that enzyme activity depends on changes in substrate and product concentrations. Beyond this, though, different compounds can exert activatory or inhibitory effects on enzymes. These modifiers are not directly involved in catalysis as substrates or products but are able to alter enzyme activity by changing binding constants or turnover rates. The modifiers are metabolites that are products of reactions located up- or downstream of the target reaction or in competing pathways within the metabolic network. One example of an important modifier in the metabolic network of many organisms is fructose 1,6-bisphosphate (FBP). FBP is produced during glycolysis by the enzyme phosphofructokinase and has been found to activate or enhance the activity of some other metabolic enzymes, such as lactate dehydrogenase (LDH) of *Streptococcus mutans* [2] and pyruvate kinase (PYK) in yeast [3]. Depending on how a modifier affects an enzyme, different types of enzyme regulation can be distinguished. One type of enzyme regulation is allosteric regulation in which the modifier binds to the enzyme at a site distinct from the active site and thereby changes its catalytic properties in an activatory or inhibitory manner. Allosteric enzymes are often found at key positions in metabolic pathways, due to their high sensitivity to small changes in metabolite concentrations. This high sensitivity is often accompanied by very complex kinetic mechanisms [4].

In glycolysis, one important allosterically regulated enzyme is PYK [3]. PYK catalyses the last step of this pathway and is generally assumed to be irreversible under physiological conditions. PYK is responsible for the transfer of a phosphate moiety from phosphoenolpyruvate (PEP) to ADP producing ATP and pyruvate (PYR). PYR serves as a branch-point or linker for many metabolic pathways. Thus, PYK links glycolysis, the primary energy metabolism, to cellular metabolism.

Lactic acid bacteria (LAB) rely almost exclusively on glycolysis for their energy production under anaerobic conditions, which reinforces the key role of PYK in their metabolism [5]. These organisms are closely related, but have adapted to a huge variety of different native environments. Some LAB are used in the food industry, especially in the dairy industry, and are present in human and animal digestive tracts. Others are known to be serious pathogens. The degree of virulence of many LAB is assumed to be reflected in their metabolism [6] due to their adaptation to different environments. Consequently, we have studied the regulation of PYK in LAB. Experimental studies on the PYKs of many LAB have revealed inhibition by inorganic phosphate (Pi) [7]–[10]. Furthermore Levering *et al.* recently reported a direct coupling between the intracellular Pi level and the glycolytic flux [11]. It was observed that the intracellular Pi concentration drops at high glycolytic flux, when Pi is simultaneously incorporated into sugar-phosphates. The decrease of the Pi level leads to a reduction of the PYK inhibition and the simultaneous activation of PYK by increasing levels of sugar-phosphates [11], [12]. In contrast to the rather conserved Pi inhibition of PYK in LAB, the activation of PYK shows high variability regarding the type of activating compound [7], [13]. The variation in the activators might reflect or originate in minor metabolic differences in the LAB due to their adaptation to different native environments.

The aim of this study is to predict the potential activators of the PYKs of three closely related LAB, namely *Streptococcus pyogenes*, *Enterococcus faecalis* and *Lactobacillus plantarum*, which are adapted to different native environments. We therefore combined comparative structural analysis and kinetic modelling in order to predict and validate potential allosteric activators for the target PYKs. The methods applied were tested for well-studied PYKs, particularly that of *Lactococcus lactis*, and then applied to the target PYKs. We first analysed the amino acid sequences and three-dimensional structures of the different PYKs. The structures of the LAB PYKs that had not been solved crystallographically were derived by comparative modelling. We compared the binding of phosphate moieties, the electrostatic properties of the allosteric activator binding sites, and the binding poses of six different potential activators computed with a molecular docking tool. The set of six potential activators was selected based on known activators of other PYKs and all the activators were sugar-phosphate compounds. The docking solutions were compared to those for PYKs with known allosteric activators in order to predict allosteric ligands for enzymes with unknown allosteric activators. To consider the physiological context of each of the LAB PYKs, the predictions were then evaluated using a kinetic model for PYK. This combined computational approach employing molecular and kinetic modelling, enabled us to predict the allosteric activators for the PYKs from *Streptococcus pyogenes*, *Enterococcus faecalis* and *Lactobacillus plantarum*.

## Results/Discussion

For the structural analysis of the allosteric activator binding sites of the PYKs from *Lactococcus lactis, Streptococcus mutans*, *Streptococcus pyogenes*, *Enterococcus faecalis* and *Lactobacillus plantarum*, structures of the enzymes were generated by comparative modelling. The template structure for modelling had an open allosteric site and consisted of a chimera of the crystal structures of the PYKs from *Bacillus stearothermophilus* (PDB id: 2E28) and *Escherichia coli* (PDB id: 1PKY) [14], [15]. The crystal structures of the PYKs from *Saccharomyces cerevisiae* (PDB id: 1A3W) and *Homo sapiens* (PDB id: 3GR4) were selected as additional reference structures for the structural comparison [16].

### PYKs show a conserved phosphate binding site in the allosteric activator binding site

**Figure 1:**
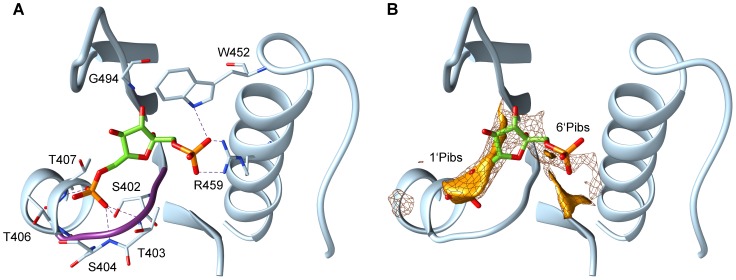
Allosteric binding site of PYK with the activator FBP bound. Residues V400 to V411, K446 to F470 and S481 to Q496 of the crystallographically resolved *Saccharomyces cerevisiae* PYK (PDB id: 1A3W, chain A) are shown in cartoon representation. Panel A shows possible hydrogen bonds between the FBP phosphate groups and the residues of PYK within a distance of 3 Å (all in stick representation). The structural P-loop motif (STSG) is coloured in purple. Panel B illustrates the phosphate interaction sites computed with the GRID program. The binding site of the 1′-phosphate moiety of FBP in the allosteric site is referred to as 1′Pibs and that of the 6′-phosphate moiety of FBP as 6′Pibs. The interaction energy is displayed at isocontours of −10 kcal/mol (mesh surface) and −13.5 kcal/mol (solid surface). Only the phosphate interaction sites that are located within 6 Å around FBP are shown.

Allosterically regulated PYKs are generally activated by a compound composed of one sugar moiety and at least one phosphate moiety [9], [13], [17]–[19]. Phosphate moieties are known to play an important role in the allosteric activation of yeast PYK. We therefore analysed the interactions between phosphate moieties and the activator binding site of PYKs with the GRID program. GRID was used to compute the energetically most favourable binding sites for a phosphate probe [20]–[24]. The FBP-activated reference PYKs from *Saccharomyces cerevisiae* (1A3W) and *Homo sapiens* (3GR4) show two strong interaction regions for the phosphate probe in the activator binding site. The interaction sites coincide with the positions of the phosphate moieties of FBP in both crystal structures. Similar phosphate interaction sites were observed for the crystal structure of *Escherichia coli P*YK (1PKY) which was solved without ligand present although *Escherichia coli* PYK is known to be FBP-activated [17]. Figure 1 shows the allosteric site of *Saccharomyces cerevisiae* PYK (1A3W) with FBP bound in the activator binding site and the computed phosphate interaction sites. The phosphate interaction sites that coincide with the 1′-phosphate and the 6′-phosphate moieties of FBP are labelled 1′Pibs and 6′Pibs, respectively. The computed binding energies of a phosphate group at the two sites, 1′Pibs and 6′Pibs, in all the protein structures studied are given in Table S1. The 1′Pibs site is energetically favourable for a phosphate group in all the proteins, whereas for all the LAB PYKs, the 6′Pibs site is not: the phosphate probe only has very weak binding affinities which are above a previously reported threshold value of −9 kcal/mol for identifying a phosphate binding site (see Methods) [25]–[27].

To validate our results, we applied the program Pfinder to the previously analysed PYK structures [28]. Pfinder is a program that detects phosphate interaction sites based on structural motifs of known phosphate binding sites. With this program, we were able to reproduce the results for the 1′Pibs site, but failed for the 6′Pibs, due to the lack of a known phosphate binding motif at the 6′Pibs site.

**Figure 2:**
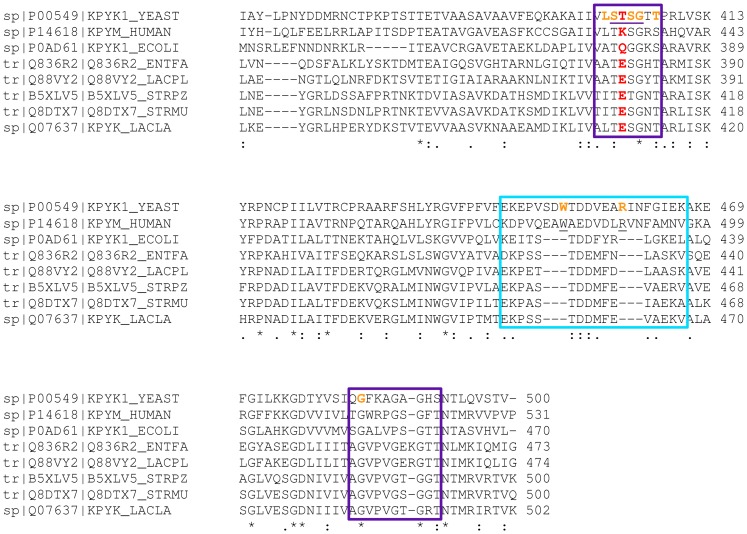
Section of the multiple sequence alignment of PYK showing the C-domain with the allosteric site. The boxed sequences directly contribute to the allosteric binding site. The residues in the purple boxes contribute to the phosphate binding site referred to here as 1′Pibs and those in the cyan box to 6′Pibs. The residues underlined in purple within the 1′Pibs site form a structural P-loop motif as discussed by Hirsch and colleagues [30]. The residues marked in orange correspond to the residues that interact with the allosteric ligand, FBP, in the *Saccharomyces cerevisiae* PYK (1A3W). The LAB PYKs show a conserved glutamate residue at the center of the allosteric site highlighted in red. In *Saccharomyces cerevisiae* PYK, it was shown experimentally that the mutation of T403 to E403 prevents allosteric activation of this PYK.

The 1′Pibs site shows a structural P-loop motif (GXXX with backbone atoms positioned to interact with the phosphate group [29]) and has some similarity to a mononucleotide binding motif (GXXXXGK(S/T)) in proteins, as reviewed by Hirsch and colleagues [30]. Phosphate binding motifs are in general very variable in different protein structures [31]. Hirsch and colleagues performed a statistical analysis of the amino acid pattern and the phosphate binding properties of the phosphate binding sites of crystallographically solved protein structures that allowed the identification of 4 different patterns [30]. The properties of the 1′Pibs of PYK coincide with one of these patterns, showing a high abundance of the amino acids glycine, threonine, serine and alanine, as well as a high number of hydrogen bonds between the phosphate moiety and the protein backbone as can be seen in Figures 1 and 2. Similar results were observed by Copley and Barton, who analysed the abundances of amino acids for structurally different phosphate binding sites [31].

### Lactic acid bacteria PYKs lack a second phosphate binding site in the activator binding site

In contrast to the 1′Pibs, the 6′Pibs shows higher variability and no phosphate binding motif. In the yeast and the human M2-type PYKs, the side chains of tryptophan (W452 in 1A3W and W482 in 3GR4) and arginine (R459 in 1A3W and R489 in 3GR4) residues are computed to interact with the 6′-phosphate moiety of FBP (see Figure 1). Both the tryptophan and the arginine residues are missing in the bacterial PYKs. In the PYK from *Escherichia coli*, an interaction of the 6′-phosphate moiety of FBP and the residue R431 might be possible, but no such interacting residues are observed for the PYKs in the LAB. Although this might indicate preferred activation by sugar monophosphates, PYK activation by FBP is reported for some LAB PYKs, including those from *Lactococcus lactis, Lactobacillus delbrueckii, Streptococcus mitis* and *Streptococcus sanguinis* [7], [9], [32]. The interaction strengths computed for the phosphate probe with the GRID program, which does not rely on structural motifs, are much weaker than for the *Saccharomyces cerevisiae* and the human PYKs at both phosphate binding sites (see Table S1).

One reason for the weak phosphate binding in the LAB PYKs might be the presence of a negatively charged glutamic acid residue at the entrance of the allosteric binding site that might hamper the electrostatic steering of the allosteric ligand due to its phosphate moiety (Figure 2). The presence of a glutamic acid residue at this position was reported as an important property of unregulated PYK isoforms [33]. Moreover, for yeast PYK, it was experimentally shown that a mutation of threonine (T403) to glutamic acid at this structurally conserved position prohibits the allosteric activation of the enzyme by FBP [34].

Different studies on the PYKs from *Lactococcus lactis* and other LAB reported a strong allosteric activation of these PYKs by activators composed of sugar-phosphates such as FBP and glucose 6-phosphate (G6P), despite the presence of this glutamic acid residue in the centre of the phosphate binding site [7], [9], [32]. These observations appear contradictory to those for *Saccharomyces cerevisiae* PYK and to previous suggestions about the properties of non-allosterically regulated PYK isoenzymes in mammals [34], [35].

### The sugar moiety is critical for activator binding in the lactic acid bacteria PYKs

**Figure 3:**
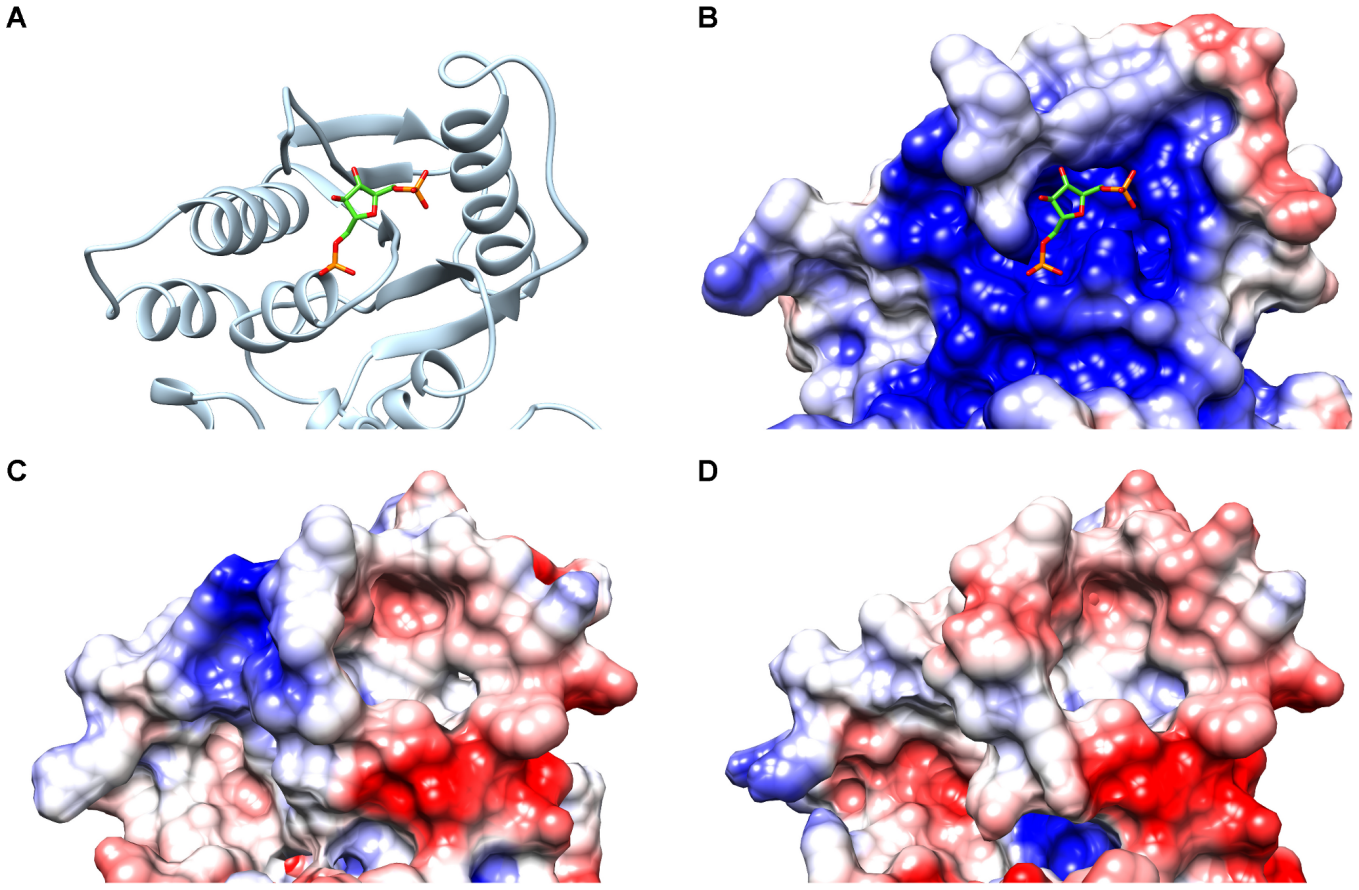
Electrostatic potentials at the allosteric sites of PYKs. The electrostatic potential is mapped on the molecular surface of each enzyme. Negative potentials are displayed in red, positive potentials in blue on a scale from −2 to +2 kT/e. Panel A shows a section of the crystallographically resolved *Saccharomyces cerevisiae* PYK (PDB id: 1A3W, chain A) in cartoon representation with the activator FBP bound in the allosteric site in stick representation. The electrostatic potentials displayed in panels B to D correspond to the same regions of the structure as shown in panel A. Panel B represents the electrostatic potential computed for the *Saccharomyces cerevisiae* PYK, panels C and D show the electrostatic potentials computed for the models of PYKs from *Streptococcus pyogenes* and *Lactobacillus plantarum*, respectively. The *Saccharomyces cerevisiae* PYK displays a broad region of positive potential at the allosteric binding site, whereas in the LAB PYKs, a rather negative potential is observed in parts of the allosteric binding site and in its proximity (panels C and D). These negatively charged regions may hinder electrostatic steering of allosteric activators to the allosteric site.

We computed the electrostatic potentials of the PYKs with the program UHBD [36]. The calculated electrostatic potentials at the allosteric site reflect the previously observed differences between the LAB PYKs and the type 1 PYKs from *Escherichia coli*, *Saccharomyces cerevisiae* and the human M2-type PYK isoenzyme. The type 1 PYKs and the human M2-type PYK isoenzyme have a positive electrostatic potential at the activator binding site whereas more negative potentials are observed for the allosteric binding site of the LAB PYKs (Figure 3).

**Figure 4:**
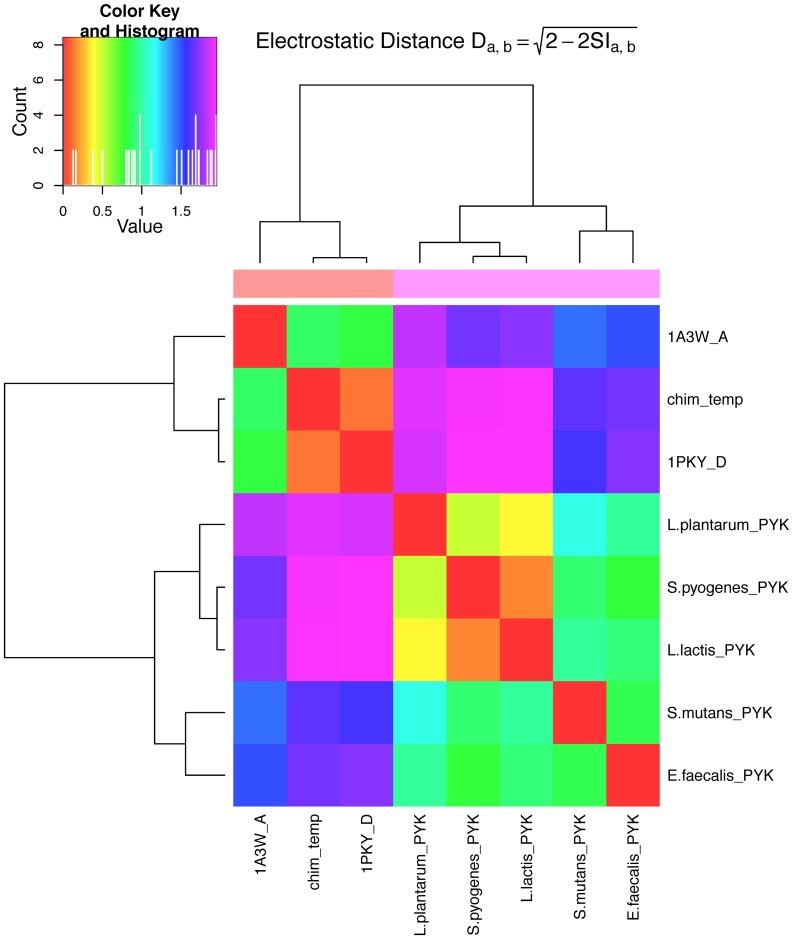
Quantitative comparison of the electrostatic potentials at the allosteric sites of PYKs. Identical and highly similar electrostatic potentials are indicated by red and orange colours. Light blue fields indicate no correlation. Anti-correlation of the electrostatic potentials is represented in pink, as shown in the colour key. Two clusters of different electrostatic potentials are observed. One includes the LAB, indicating almost identical electrostatic potentials in the allosteric sites of the PYKs from *Lactococcus lactis* (L.lactis_PYK) and *Streptococcus pyogenes* (S.pyogenes_PYK). Both also show high correlation to the allosteric site of the PYK from *Lactobacillus plantarum* (L.plantarum_PYK). Little correlation is observed to the other LAB. The second cluster is formed by the electrostatic potentials computed for the template (chim_temp) and the reference crystal structures (1PKY, 1A3W). As expected, almost identical electrostatic potentials are observed for the allosteric site of the chimeric template and the crystal structure 1PKY. The computed similarity indices are shown in Table S2.

We performed a Protein Interaction Property Similarity Analysis (PIPSA) to quantitatively compare the electrostatic potentials at the allosteric binding site of the PYKs (Figure 4) [37], [38]. Similarity of the computed electrostatic potentials can provide an indication of similarity in ligand binding [39]. We observed high similarity between electrostatic potentials at the allosteric sites for the PYKs from *Lactococcus lactis*, *Streptococcus pyogenes* and *Lactobacillus plantarum*. The electrostatic potentials computed for the PYKs from *Escherichia coli, Saccharomyces cerevisiae*, and the chimeric template showed a high similarity to each other but no similarity to the electrostatic potentials of the LAB PYKs. The PYKs from *Lactococcus lactis* and *Streptococcus pyogenes* show almost identical electrostatic potentials at the allosteric site, indicating potentially strong similarities in ligand binding for the *Lactococcus lactis* and *Streptococcus pyogenes* PYKs.

Differences in the electrostatic potentials at the allosteric sites of the PYKs from LAB and the other PYKs, combined with the weaker interaction of the phosphate probe with the LAB PYKs indicates that the major factor determining activator binding differs between PYKs of different origin. Bond and colleagues suggested that, in the case of the PYK from *Saccharomyces cerevisiae*, the phosphate moieties of FBP play a major role in enzyme-activator complex stabilization corresponding with a strongly increased dissociation constant (K_D_) for fructose 6-phosphate (F6P) (K_D_ = 1.3 mM) and fructose 1-phosphate (F1P) (K_D_ = 0.49 mM) compared to FBP (K_D_ = 0.01 mM); these compounds differ from FBP only in the absence of the 1′- or the 6′-phosphate-moiety [34].

Although activator binding cannot be directly equated with allosteric activation, sugar-monophosphates are observed to be the preferred or the best allosteric activators in some LAB PYKs [8], [13]. As the allosteric sites of the LAB PYKs are less favourable for phosphate binding than those of the *Saccharomyces cerevisiae*, *Escherichia coli* and human PYKs, we suggest that the sugar moiety of the allosteric activator plays a relatively much more critical role in binding in LAB PYKs.

### Different sugar preferences for different lactic acid bacteria PYKs

We next considered the binding of whole ligands to the allosteric sites. A set of six compounds was selected which includes known allosteric activators of different LAB PYKs (see Table 1). We computed favourable binding poses of these ligands by docking them into the allosteric binding site of the PYK structures. The results for the reference PYKs were then compared to those for the PYKs from *Streptococcus pyogenes*, *Enterococcus faecalis* and *Lactobacillus plantarum*, to predict allosteric activators for them. The PYKs of *Escherichia coli*, *Streptococcus mutans* and *Lactococcus lactis* were selected as reference enzymes because these enzymes have already been studied extensively and allosteric activators have been reported (Table 1).

**Table 1:** Experimentally determined allosteric activators of PYKs*.

Organism	Modifier					References
	*Activation*		*No Activation*	*Inhibition*		
	ligand	k_0.5v_ [mM]		ligand	k_0.5v_ [mM]	
*Escherichia coli*	FBP	0.04	G6P, F6P	-	-	[17], [76]
*Streptococcus*	G6P,	0.04	F6P	Pi	-	[19]
*mutans*	(R5P)$	0.41				
*Lactococcus*	G6P,	0.012	-	Pi	1.3	[13], [32]
*lactis*	R5P,	0.015				
	F6P,	0.03				
	FBP,	0.057				
	Gal6P	0.45				

* k_0.5v_ values are dependent on the experimental conditions. See references for further information.

$ partial activation by R5P.

The docking of the ligands and the computation of the most favourable binding poses was done with the program GLIDE implemented in the Maestro software of Schrödinger [40]–[42]. 20 docking solutions per ligand were computed and the best pose was chosen using the Emodel docking score. (The PYK structures and the corresponding ligands with the best binding poses are provided in supplementary Dataset S1.) The selected binding poses of the different ligands were compared using the Emodel docking score: the more negative the score, the more favourable the binding of the ligand was predicted to be. We ranked the ligands according to the most negative Emodel docking scores and compared the ranking to the experimentally determined ligand ranking (Table 1). The Emodel docking score is composed of the non-bonded interaction energy, the Glide Score (computed binding free energy) and the ligand strain energy. The Glide Score computed free binding energies were very similar for all ligands and all PYK structures examined and did not allow reproduction of the experimentally observed ligand ranking, whereas the Emodel score did although its magnitude bears no relation to the experimental binding affinities or allosteric activities. The docking solutions for each ligand were clustered into groups of similar binding poses based on the ligand's pose and orientation. We considered ligand binding to be more entropically favoured the more different binding poses were present.

For the crystal structure of *Escherichia coli* PYK, the most favoured ligand for binding in the allosteric site, FBP, was computed to have an Emodel score of −91.7 kcal/mol. The docked positions of the phosphate groups of FBP coincide with the phosphate interaction regions computed with the GRID program. Experimentally, FBP was shown to be the sole allosteric activator of this PYK. No activation was observed for G6P, F6P or F1P (Table 1). Consistently, all other ligands show binding poses with docking scores that are less favourable by more than 10 kcal/mol and a reduced probability to bind in the allosteric site (see Table S3).

The *Streptococcus mutans* PYK is activated by G6P [19]. According to the docking, the most favoured ligands for binding are galactose 6-phosphate (Gal6P) and G6P with Emodel docking scores of −60.7 and −60.5 kcal/mol, respectively. As far as we know, Gal6P has not been tested experimentally as an activator of the *Streptococcus mutans* PYK. The computation of G6P as an activator is in agreement with previous reports [8], [19]. The docking scores for the ligands F1P, FBP and F6P are close to that for G6P (see Table S4). However, the number of docking solutions where the ligands are bound outside the allosteric site is higher indicating that the probability of binding in the allosteric site is lower, and suggesting that none of these ligands are good activators. Indeed, FBP and F6P were shown experimentally not to be activators of this PYK [19]. No information about F1P was found.

The *Lactococcus lactis* PYK was shown experimentally to be activated by various different sugar-phosphate ligands [13]. The strongest allosteric activators were reported to be G6P (ka_0.5v_ = 0.012 mM) and R5P (ka_0.5v_ = 0.015 mM). A slightly lower activation level was reported for F6P (ka_0.5v_ = 0.03 mM), FBP (ka_0.5v_ = 0.057 mM) and Gal6P (ka_0.5v_ = 0.45 mM) (Table 1). The docking scores of the ligand poses resulted in the same ligand ranking order except for Gal6P. The most favourable ligand pose was computed for G6P with −57.1 kcal/mol. A similar score was computed for the ligand pose of Gal6P at −54.1 kcal/mol, although Gal6P was found experimentally to be one of the weakest activators of this PYK. All the other ligands were computed to bind less favourably (see Table S5). Gal6P differs from G6P only in the orientation of the C4′-hydroxyl group. It is possible that the C4′-hydroxyl group of G6P contributes not only to binding at the allosteric site but also to the subsequent allosteric activation mechanism.

Almost all the docking solutions for the *Lactococcus lactis* and *Streptococcus mutans* PYKs resulted in ligand orientations in which the phosphate moiety either occupies or points in the direction of the 6′Pibs, the weaker phosphate binding site. The binding of the allosteric activator is observed to be mainly maintained by hydrogen-bonds between the sugar-moiety and the protein (see Table 2). These results confirm our hypothesis that phosphate binding is less important for activator binding in the LAB PYKs. The docking solutions for the *Escherichia coli* PYK, on the other hand, show a strict orientation of the phosphate-moiety towards the 1′Pibs, the stronger phosphate binding site. In the case of FBP, the allosteric activator binding *Escherichia coli* PYK, hydrogen bonds seem to be preferentially formed between the phosphate-moieties and the protein, but are also found to be possible between the sugar-moiety and the protein.

**Table 2:** Possible hydrogen bonds between the PYK structures and FBP, G6P and R5P*.

	FBP		G6P		R5P	
Organism	*Pi-moieties*	*sugar-moiety*	*Pi-moiety*	*sugar-moiety*	*Pi-moiety*	*sugar-moiety*
*Escherichia*	Q379$	G454	Q379	A377	Q379	A377
*coli*	K382	A377	Q380	G454	G380	G454
	G380		S383	V457	K382	V457
	S383		K382		S383	
	T401					
*Streptococcus*	F401	G454	F401	E379	-	I377
*mutans*				G454		E379
				S459		S459
				G461		
*Lactococcus*	E381	V457	-	L379	-	T459
*lactis*	G456	V459		G456		I377
	T461	G460		T461		
				R463		

* possible hydrogen bonds are computed with UCSF Chimera version 1.6.2.

$ hydrogen bonding between the oxygen linking the phosphate moiety and the sugar moiety.

We correctly predicted the allosteric activators for all three reference PYKs and therefore applied the same approach to the *Streptococcus pyogenes*, *Enterococcus faecalis* and *Lactobacillus plantarum* PYKs to predict potential allosteric ligands for these PYKs.

**Figure 5:**
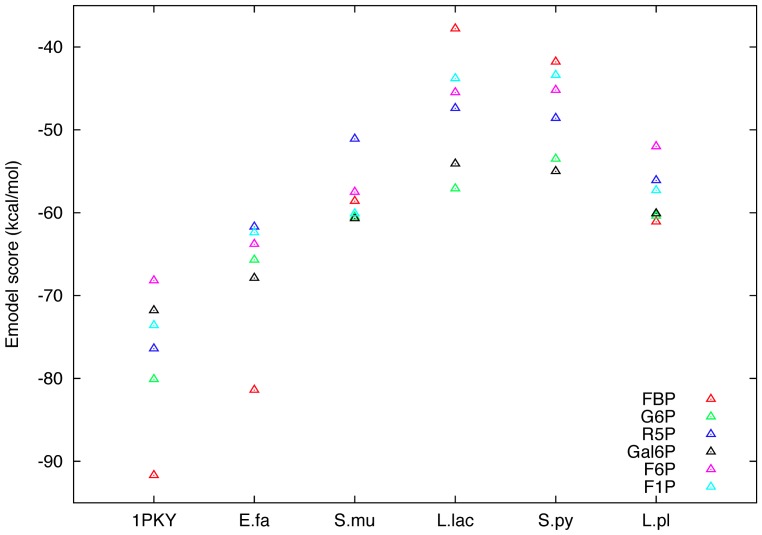
Computed Emodel scores of the most favourable ligand binding poses docked in the allosteric sites. Similarities in the scores of the binding poses (E_model_) are observed for the PYKs of *Escherichia coli* (1PKY) and *Enterococcus faecalis* (E.fa) with FBP being the most favourable ligand for binding. The scores for the other ligands are more than 10 kcal/mol less favourable. Similarities in the scores are also found for the PYKs of *Streptococcus mutans* (S.mu), *Lactococcus lactis* (L.lac), *Streptococcus pyogenes* (S.py) and *Lactobacillus plantarum* (L.pl) which show overall weaker ligand binding with more similar scores for the different ligands. A high similarity between the scores of the *Lactococcus lactis* and *Streptococcus pyogenes* PYKs is observed.

**Figure 6:**
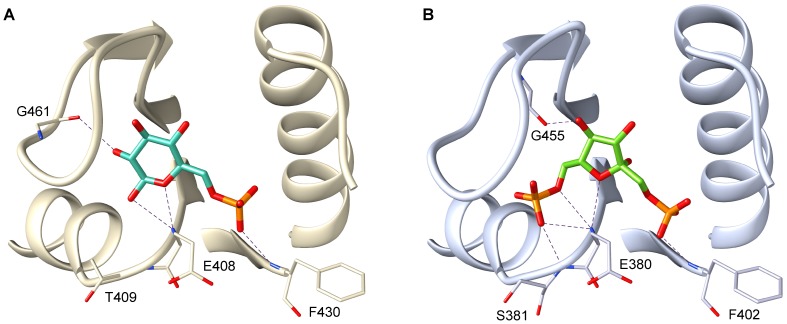
Docking poses of the ligands with the most favourable interaction energies for two modelled PYKs. (A) G6P in the allosteric site of the *Streptococcus pyogenes* PYK. (B) FBP in the allosteric site of the *Enterococcus faecalis* PYK. The protein allosteric sites are shown in cartoon representation and the potential allosteric activators are shown in stick representation. The predicted allosteric activator FBP is bound in the allosteric site in its most favourable binding pose with a score of −81.4 kcal/mol. Hydrogen bonds between the ligands and the proteins (with a maximum length of 3.2 Å) are indicated by dashed lines.

The results of PIPSA showed a high similarity between the allosteric binding sites of *Lactococcus lactis* and of *Streptococcus pyogenes* PYKs. The docking results were also similar (Figure 5). For *Streptococcus pyogenes* PYK, the scores for the most favourable binding poses of Gal6P and G6P were −55.0 and −53.5 kcal/mol, respectively. Figure 6 shows the *Streptococcus pyogenes* PYK with G6P docked in the allosteric site in its most favourable binding pose. The computed scores for all ligands and their ranking according to binding pose are highly similar to the results for *Lactococcus lactis* PYK (see Table S6). Therefore, we anticipate that, as for *Lactococcus lactis* PYK, *Streptococcus pyogenes* PYK can be activated by a variety of different ligands with Gal6P and G6P being among the strongest activators.

The docking results for the *Lactobacillus plantarum* PYK showed similar scores for 3 ligands: FBP with −61.1 kcal/mol, G6P with −60.4 kcal/mol, and Gal6P with −60.1 kcal/mol (Figure 5). The most favourable scores of binding poses computed for the other ligands are also very close. There are again similarities to the results for *Lactococcus lactis* PYK, suggesting that the *Lactobacillus plantarum* PYK can be activated by a variety of different ligands.

The docking results for the *Enterococcus faecalis* PYK showed similarity to the results for the *Escherichia coli* PYK (Figure 5). The most favourable binding pose was computed for FBP with −81.4 kcal/mol (Figure 6). The scores for the other ligands are very close to each other, but are more than 13 kcal/mol less favourable than the one for FBP, thus show a very similar trend in the computed binding poses to that observed for the *Escherichia coli* PYK. Therefore, we predict FBP to be the most probable and sole activator for this PYK.

In summary, two different binding patterns are observed (Figure 5): i) one favourable binding pose for one ligand, the allosteric activator. In this case, observed for the *Escherichia coli* PYK and the *Enterococcus faecalis* PYK, we identified FBP to be the most favourable and sole allosteric activator. ii) more similar binding poses with poorer scores accompanied by reduced activator specificity. This case was observed for the *Lactococcus lactis*, *Streptococcus pyogenes* and *Lactobacillus plantarum* PYKs and partially for the *Streptococcus mutans* PYK. The reduced specificity for the allosteric activators was experimentally shown for the *Lactococcus lactis* PYK. Similar results are predicted for the *Streptococcus pyogenes* PYK and for the *Lactobacillus plantarum* PYK. The *Streptococcus mutans* PYK does not share the weak specificity for the allosteric activators, with G6P and R5P being the sole activators, as found in the *Lactococcus lactis* PYK. To the best of our knowledge, only information about the PYK activation but no information about the binding of other ligands to the allosteric site has been reported for the *Streptococcus mutans* PYK, as observed for instance for the *Lactobacillus bulgaricus* PYK [43].

### The predicted activators match the physiological context of the organisms

We next analysed the role of the potential activators in their physiological context. We generated a kinetic model for PYK that reflects its regulation on the metabolic level. This model allows us to investigate the potential activators, considering intracellular metabolite concentrations. (An SBML file of the kinetic model is provided in supplementary Model S1.)

**Figure 7:**
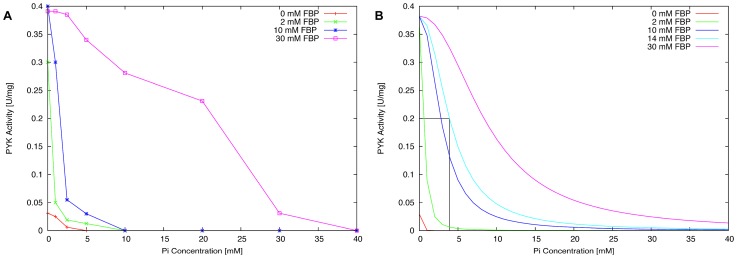
Enzymatic activity of *Lactococcus lactis* PYK at various concentrations of Pi and FBP. Panel A shows experimental measurements of the PYK activity at an increasing Pi concentration with 0 mM, 2 mM, 10 mM and 30 mM FBP present as reported by Mason *et al.* [12]. Panel B shows the simulation results of the PYK kinetic model. The kinetic model is able to predict the PYK activity for 0 mM, 2 mM and 10 mM FBP, but somewhat underestimates the activity for 30 mM FBP. According to this model, about 14 mM FBP is required to achieve the half maximum PYK activity (0.2) at a physiological Pi concentration of 4 mM (see text for details).

The kinetically well-studied *Lactococcus lactis* PYK was chosen as the reference PYK for the kinetic modelling [10], [32]. Its enzyme activity can be regulated by strong inhibition by Pi and by a set of different activators of which it is assumed that FBP dominates *in vivo* [10], [13], [32]. Both Pi inhibition and allosteric activation by FBP are described by the kinetic model. The PYK kinetics are represented by a reversible Hill equation [44]. Unknown kinetic parameters were fitted by using parameter estimation with the software COPASI [45]. For this purpose, we used the experimental data of Mason *et al.* who measured the effect of increasing Pi and FBP concentrations on the *Lactococcus lactis* PYK activity [12]. We tried different types of modifier interactions in the model: i) the modifiers bind mutually exclusively (a competitive formulation), ii) the modifiers bind independently of each other, and iii) a general formulation that allows the modifier to bind at separate sites at the same time [44]. However, only competitive inhibition was able to fit the experimental data well (Figure 7).

Levering and colleagues reported a coupling between the glycolytic flux and the intracellular Pi concentration for *Lactococcus lactis* [11]. Variations in the intracellular Pi level were found to be accompanied by changes in the glycolytic flux. Under conditions of no glycolytic flux, the intracellular Pi level was experimentally observed to reach a concentration of more than 20 mM [12], [46]. No activating compounds were detectable and PYK seemed to be completely inhibited. At high glycolytic flux, the intracellular phosphate concentration was observed to drop to about 4 mM [12], [46]. At this Pi concentration, our simulations predict a requirement of about 14 mM FBP to overcome the phosphate inhibition and to obtain half maximum PYK activity (*in vitro*) (Figure 7). This FBP concentration might be slightly overestimated in the model, but experimental measurements revealed intracellular FBP concentrations reaching 20 mM and even higher, depending on the amount of available glucose in the environment of *Lactococcus lactis* [11], [12], [46]. Other sugar-phosphate intermediates, such as G6P and F6P, are only present intracellularly at approximately 14- to 4-fold lower concentrations, thereby suggesting that FBP is physiologically the most relevant allosteric activator of *Lactococcus lactis* PYK [11], [12] for these environmental conditions.

Our activator predictions, as well as experimental observations, revealed a rather weak specificity of the *Lactococcus lactis* PYK regarding the allosteric activator [13]. The acceptance of many sugar-phosphate compounds as activators might allow the strong Pi inhibition to be overcome by the sum of the single activator concentrations. This might allow partial PYK activity to be maintained under decreasing glycolytic flux and an increasing Pi concentration.

No experimental data about allosteric regulation of *Streptococcus pyogenes* PYK are available. The high sequence identity of 77% between the PYK sequence of *Streptococcus pyogenes* and *Lactococcus lactis*, as well as the high degree of residue conservation at the allosteric site and the active site in these enzymes, together with the computed high similarity of the electrostatic potentials and ligand docking results, lead us to the conclusion that these PYKs share similar enzyme kinetics (supplementary Figure S9). Therefore, we used the kinetic PYK model of *Lactococcus lactis* to study the activator predictions of the *Streptococcus pyogenes* PYK in a physiological context. Levering and colleagues described a similar coupling of the intracellular phosphate concentration and glycolysis in *Streptococcus pyogenes* to that in *Lactococcus lactis* [11]. Measurements of the intracellular glycolytic intermediates revealed similar FBP and G6P levels to those observed in *Lactococcus lactis* with an about 4-times higher FBP level. Therefore, for *Streptococcus pyogenes* PYK, FBP is expected be the most relevant activator, even though G6P shows a more favourable binding pose. Due to different phosphate transport systems and different phosphate levels in the native environment of *Streptococcus pyogenes*, it is possible that the intracellular phosphate concentration in glycolytically active cells is slightly lower than in *Lactococcus lactis* [11]. The property of PYK to be able to be activated by a mixture of different activators seems again to be beneficial and strengthens the role of the other computationally predicted activators.

Both *Lactococcus lactis* and *Streptococcus pyogenes* reside in highly variable environments. *Streptococcus pyogenes* is a well-known human pathogen that is observed to cause infections in many different tissues ranging from the skin to the respiratory tract [47]. *Lactococcus lactis*, in contrast, is non-pathogenic, and found in many different environments such as the digestive tract of humans or animals, milk and other dairy products [48]. These highly variable environments demand a high metabolic flexibility of the organisms to adapt to changing conditions. The metabolic flexibility is reflected in the diversity of phosphorylated sugar compounds in the cell. A reduced specificity for the allosteric activator of PYK seems to be beneficial. It allows the organisms to transfer the information that sugars are available to PYK and that glycolysis can proceed. One example for changing environmental conditions is the acidification of the environment by high lactate production which leads to a decrease of the intracellular pH in *Lactococcus lactis*. The low intracellular pH was reported to negatively affect metabolic enzymes [49]. Changes in the enzyme activities are shown to strongly vary the pools of the glycolytic intermediates. In this context, Even and colleagues measured high G6P concentrations whereas FBP was present at about ten times lower concentrations, thereby suggesting that under this conditions of low intracellular pH, G6P is physiologically the most relevant allosteric activator of *Lactococcus lactis* PYK [49].

In *Enterococcus faecalis*, which in contrast resides in a very rich environment, similar intracellular phosphate concentrations at high glycolytic flux to those in *Lactococcus lactis* are assumed, due to the presence of functionally similar phosphate transport systems to that found in the genome sequence of *Streptococcus pyogenes*. The intracellular FBP concentrations are high, and are assumed to be similar to those observed for *Lactococcus lactis* [50]. This supports FBP being the physiologically most relevant allosteric activator. It also coincides with the computational prediction of FBP as the sole allosteric activator and the high specificity of the allosteric activator binding site for this ligand.

For *Lactobacillus plantarum*, only a few data are available. Experimental measurements of the intracellular metabolite levels revealed a similar situation to that observed for *Lactococcus lactis* [51]. The FBP level is very high and was measured to be 17 mM, whereas other sugar-phosphate levels, such as those of G6P and F6P, were found to be about ten-fold lower. This suggests that FBP is again the most relevant allosteric activator of this PYK, although very similar binding poses are computed for the other potential allosteric ligands.

PYKs are found in all species ranging from bacteria up to mammals with only a few exceptions [52]. Analysis of the PYK sequences revealed a high degree of conservation within the active site responsible for PEP and ADP binding [33], [34] (supplementary Figure S1). The allosteric binding site shows a much higher variability, even for very closely related organisms. This variability might be caused by changes in the metabolism of the species due to their adaptation to different native environments. Further, PYKs with different allosteric properties have developed. Based on the two co-existing PYKs in *Escherichia coli*, 2 different PYKs with different allosteric properties can be distinguished in bacteria [33]: i.) the type I PYK, allosterically activated by FBP, and ii.) the type II PYK, allosterically activated by AMP and R5P. Sequence analysis of the PYKs from the LAB analysed here revealed high similarity to PYKs of type I and to the type I PYK of *Escherichia coli*. Table 3 shows the pairwise sequence identities among the PYKs discussed in this study. The allosteric activators of this type are again found to be very variable. Different sugar-phosphates are experimentally observed for different PYKs with different degrees of activator specificity. Interestingly, the PYKs from *Lactococcus lactis* and *Streptococcus mutans* share a sequence identity of 79%, but differ significantly in their allosteric regulation (Tables 1, 3). The PYK of *Streptococcus mutans*, for instance, shows a high specificity for G6P and is only partially activated by R5P but not by FBP or F6P [19]. The PYK of *Lactococcus lactis*, in contrast, shows very weak activator specificity and can be activated by FBP, G6P, F6P, R5P, Gal6P and other sugar-phosphate compounds [13]. This weak specificity against the allosteric activator can have a beneficial effect. In *Lactococcus lactis* and *Streptococcus pyogenes*, we assume that the mixture of possible activators lowers the threshold to overcoming the phosphate inhibition. More importantly, weak activator specificity might provide an increased metabolic flexibility for the organisms. Residing in diverse environments requires frequent adaptation to carbon sources other than glucose. In milk, for instance, *Lactococcus lactis* is mainly faced with lactose as a carbon source; lactose is a disaccharide composed of glucose and galactose. The presence of different carbon sources and different growth media change the relative composition of phosphorylated sugar compounds and might therefore affect their impact on PYK activation and therefore increase the organisms flexibility to adapt to different conditions [53].

**Table 3:** Pairwise sequence identities among the PYKs from different organisms.

[%]	S.mutans	S.pyogenes	L.plantarum	E.faecalis	E.coli	S.cerevisiae	H.sapiens
L.lactis	79	77	51	46	39	38	38
S.mutans		86	49	44	42	39	39
S.pyogenes			50	46	40	38	38
L.plantarum				64	45	41	40
E.faecalis					51	41	43
E.coli						44	48
S cerevisiae							52

Weak specificity for the allosteric activators can also have negative effects. Bras and Garel have experimentally studied the allosteric activation of the PYK from *Lactobacillus bulgaricus* [43]. This PYK was found to be activated strongly by G6P and R5P and slightly less strongly by F6P and 2-desoxy-G6P. Moreover, AMP was observed to be a weak activator. A mixture of the weak activator, AMP, with any of these strong activators resulted in a strong inhibition of the PYK activity, suggesting that the weak allosteric effector competes with the strong allosteric effectors for the activator binding site. Similarly, FBP was found to be a strong inhibitor of this PYK.

Experimental data about the allosteric activation of the PYK from *Lactococcus lactis* revealed that all the activators studied by Thomas are strong activators with little difference in their activation efficiency [13]. Therefore, we assume no competitive inhibitory effects between the single activators for the PYKs of *Lactococcus lactis* or *Streptococcus pyogenes* and probably also *Lactobacillus plantarum*.

### Concluding remarks

LAB play an important and diverse role in human health. Many LAB are harmless microorganisms that have, for example, been used for food fermentation processes for hundreds of years. Others are multi-resistant pathogens. The reasons why LAB become pathogenic are still a matter of debate. In this study, we selected three representatives of closely-related LAB, namely *Lactobacillus plantarum*, *Streptococcus pyogenes* and *Enterococcus faecalis*. *Lactobacillus plantarum* is widely used in the dairy industry. *Streptococcus pyogenes* is a well-known pathogen, causing scarlet fever, whereas *Enterococcus faecalis* is known to cause serious hospital-acquired infections, but also contributes to the special taste of cheese.

Ongoing comparative studies aim at a better understanding the differences between these LAB, focusing on the LAB metabolism [11], [54]. Investigation of the metabolism might provide novel strategies for medication and is also interesting for industrial purposes. Together with *Lactococcus lactis*, the genomes of all three LAB have been fully sequenced which allows comparative studies at different levels of detail.

To understand the metabolic behavior of the organisms, we need to understand the dynamics of the metabolic system, especially upon environmental changes. PYK plays a key regulatory role in the central metabolism of LAB. So far, there are no experimental data on the enzyme kinetics or on the regulation of the PYKs from *Lactobacillus plantarum*, *Streptococcus pyogenes* and *Enterococcus faecalis*. The regulation is particularly important for the dynamics of the metabolic network. Therefore, we used a novel combination of computational methods to predict the activators of these enzymes. Supplementary figure S2 shows the workflow. In each step, we used experimentally well-studied PYKs as references, namely, the human M2-type PYK, or the PYK from *Saccharomyces cerevisiae*, *Escherichia coli*, *Streptococcus mutans* or *Lactococcus lactis*. We applied comparative modeling to generate three dimensional structures of the LAB PYKs and compared those to the reference structures. We analyzed phosphate binding motifs at the allosteric site with the program Pfinder, studied the interaction energies between a phosphate probe and the allosteric site using the GRID program, computed the electrostatic properties with the program UHBD and compared the electrostatic properties at the allosteric sites quantitatively using PIPSA. We found that the LAB PYKs differ from the other PYKs, showing only one weak phosphate binding site at the 1′Pibs and less pronounced negative potentials at the allosteric sites. We suggest that the binding of the phosphate moiety of the potential activators is not the most important factor for the allosteric activation.

Next, we used a docking approach to study activator binding in comparison to experimentally observed activator constants k_0.5v_ and predicted allosteric activators for the LAB PYKs. We further observed differences in the activator binding specificity. The PYK of *Enterococcus faecalis* strongly favors the binding of FBP that seems to be the sole activator. The PYK of *Streptococcus pyogenes*, in contrast, does not show a strong preference for one ligand and seems to be activated by a set of different allosteric activators. To find the physiologically most relevant activator, we generated a kinetic model of the *Lactococcus lactis* PYK and simulated the enzyme activity at physiological activator and inhibitor concentrations. By using this information we could show that the activator with the best activation constant is not always physiologically the most relevant activator. We applied this strategy to the PYKs of *Streptococcus pyogenes*, *Lactobacillus plantarum* and *Enterococcus faecalis* and studied the docking results with respect to the experimentally determined intracellular activator concentrations of the respective LABs. FBP seems to be the physiologically most relevant activator for the LAB PYKs. The differences of the activator specificity of the LAB PYKs might relate to the adaptation to different native environments.

In future work, the analysis described here could be extended to further LAB PYKs and it could be particularly interesting to examine those PYKs for which not only the allosteric activator but also weak or non-activatory ligands are reported. This study might help to identify important structural ligand properties for binding or for the allosteric activation.

The techniques used in this study are well-established computational methods with various applications. We used these methods in a novel combination to predict allosteric regulators of closely related enzymes.

The combination of structure-based and kinetic modelling employed here can readily be applied to other enzymes and we anticipate that it will become an increasingly powerful approach in comparative systems biology projects as detailed kinetic and structural data become available.

## Methods

### Comparative modelling

As no crystal structures are available for the PYKs from *Lactococcus lactis, Streptococcus mutans*, *Streptococcus pyogenes*, *Enterococcus faecalis* and *Lactobacillus plantarum*, the three dimensional structures of these target enzymes were derived by comparative modelling using the software MODELLER [55]–[58]. All sequences were taken from the UniProt database [59]. More detailed sequence information is given in the Table S4.

#### Template selection

Possible template structures were selected with the BLASTp (protein-protein blast) tool on the NCBI webserver [60]. The “search set” was restricted to the PDB database [61] and the Position-Specific Iterated BLAST algorithm [62] was selected in combination with the BLOSUM62 scoring matrix. The algorithm parameters were set to default values.

The crystal structures of the PYKs from *Escherichia coli* (PDB id: 1PKY) and from *Bacillus stearothermophilus* (PDB id: 2E28) were selected as template structures [14], [15]. Both showed a suitably high sequence identity (over 39%) and similarity (over 60%) to the target sequences and displayed a completely resolved allosteric site in either an open conformation (1PKY) or a closed conformation (2E28). The asymmetric unit in the crystal structure of *Bacillus stearothermophilus* PYK (2E28) contains one subunit; the structure was solved to 2.5 Å and has a sulphate ion bound in the closed allosteric site. The structure of *Escherichia coli* PYK (1PKY) was solved as a homotetrameric enzyme complex in the asymmetric unit with a resolution of 2.6 Å and has no ligands in the accessible allosteric site. The subunit referred to as chain D in the 1PKY PDB file was selected for the modelling as it has the highest number of resolved amino acid residues, especially in the domain that carries the allosteric site.

All the target enzymes were modelled as monomeric structures with an open allosteric site against a chimeric template structure built from the two templates. The domain carrying the allosteric activator binding site was derived from *Escherichia coli* PYK (1PKY). The remaining structure was taken from *Bacillus stearothermophilus* PYK (2E28). The two crystal structures were superimposed in the sequence ranges T355 – I474 (2E28) and L352 – L470 (1PKY). The peptide bonds between I337 and V338 (PDB id: 2E28) and between V335 and V336 (PDB id: 1PKY, monomer D) were then selected as fusion point between the two structures. The assignment of the residues from the crystallographically solved structures, 2E28 and 1PKY, to the corresponding residues of the chimeric template is shown in the Table S5.

Two additional crystallographically resolved structures, namely for *Saccharomyces cerevisiae* PYK (PDB id: 1A3W) and human PYK M2 (PDB id: 3GR4) [16], were selected for the structural comparison of the allosteric activator binding sites. The *Saccharomyces cerevisiae* PYK structure (1A3W) was solved with a resolution of 3 Å, and shows a homodimer in the asymmetric unit with open allosteric sites and FBP bound. The human PYK structure (3GR4) has a homotetramer in the asymmetric unit with closed allosteric binding sites and FBP bound. In both cases, the chain A subunit was selected as the reference structure.

#### Sequence alignment

The target and template sequences were aligned pairwise using the MODELLER software, version 9.8, and the “alignment-append” protocol (Blundell and Sali 1993; Eswar et al. 2006; Fiser et al. 2000; Marti-Renom et al. 2000). As a control, a pairwise alignment was performed with the NCBI web server BLASTp (Basic Local Alignment Search Tool) tool based on the BLOSUM62 matrix [63].

The PYKs from *Enterococcus faecalis* and *Lactobacillus plantarum* and the template sequence from *Bacillus stearothermophilus* have an extra C-terminal domain of about 112 residues which was excluded from the alignment. The PYKs from *Streptococcus pyogenes*, *Lactococcus lactis*, and *Streptococcus mutans* contained an additional loop G12 – K40 (numbering according to the sequence of *Lactococcus lactis*) that was also not considered. The gaps due to the removed loop were not ligated but defined as additional N- and C- termini. In addition, N- and C-terminal residues with low sequence similarity were removed from the alignments in order to increase the model quality. All residues that were removed before modelling are listed in Table S6.

For the comparison of the allosteric sites, a multiple sequence alignment of all the PYK sequences analysed was performed on the EBI web server with the ClustalW2 tool [64]. The pairwise alignment and the multiple sequence alignment were computed based on the Gonnet weighting matrix [65].

#### Structure modelling

The monomeric target structures were generated by using the software MODELLER version 9.8 and the protocol “model-fast” (Blundell and Sali 1993; Eswar et al. 2006; Fiser et al. 2000; Marti-Renom et al. 2000), based on the pairwise sequence alignment. Ligands and crystallographic water sites were not considered during the modelling and were removed from the template structures beforehand. The generated models were validated with the PROCHECK tool [66].

### Computation of phosphate interaction sites

The GRID program (Goodford, 1985; Wade & Goodford, 1993; Wade, Goodford & Clark, 1993) was used to identify possible phosphate binding sites in the allosteric site for ligands with one or more phosphate moieties. To identify favourable interaction sites, a phosphate probe (HPO_4_^2−^) was used. A cubic grid was superimposed on each target structure and the phosphate probe was placed at each grid point in turn and its interaction energy with the protein was computed. The grid box extended 110 Å in each direction with a grid spacing of 0.5 Å. The computed phosphate interaction energy maps were contoured and visualized. A binding energy of −9 kcal/mol was defined as a threshold for defining a phosphate binding site. Similar threshold values were reported previously [25]–[27]. When a phosphate ion binds to a protein it must displace water molecules. The energetic cost of displacement is not included in the computed interaction energy but is approximately accounted for in the assignment of the threshold interaction energy for defining a phosphate binding site (considering that water binding sites can be defined by threshold GRID interaction energy of −6 kcal/mol which corresponds to the chemical potential of water).

In addition, we applied the program Pfinder to find phosphate binding sites [28]. This program compares a submitted protein structure with a database containing phosphate interaction motifs. We applied Pfinder to the structures *Saccharomyces cerevisiae* PYK (1A3W), *Escherichia coli* PYK (1PKY) and the modelled structures. All parameters were kept at default values.

### Electrostatic potential computation and comparison

The calculation of the electrostatic potentials of the superimposed target models and the template structures was performed with the program UHBD [36] by numerical solution of the linearized Poisson-Boltzmann equation. Polar hydrogen atoms were added to the target and template structures using WHATIF version 5.0 [67] assuming neutral pH. Changes in the residue structures and unusual protonation states introduced by the WHATIF program are given in Table S7. Additionally, the N- and C- termini of the PYK models from *Lactococcus lactis* and *Streptococcus pyogenes* introduced where the loop was removed (residues 12–40) were defined as neutral. OPLS partial charges and van der Waals radii were assigned to the atoms in the protonated structures with the UHBD program [36].

Relative dielectric constants of 78 and 2 were assigned to the aqueous medium and the protein interior, respectively. The ionic strength was set to 150 mM, to represent a physiological value [68]. The dielectric boundary was defined by the molecular surface of the proteins. The electrostatic potential was computed on a cubic grid extending 110 grid points in each direction with a spacing of 1 Å.

The comparison of the electrostatic potentials at the allosteric binding sites was done with the PIPSA (Protein Interaction Property Similarity Analysis) software [37], [38] using a sphere that included the allosteric binding site. Only the electrostatic potentials at points in the protein “skin” around the protein surface which were within the sphere volume were compared by means of pairwise Hodgkin indices. The centre of the sphere was defined as the average geometric centre of the allosteric ligand FBP as crystallized in the structure of human PYK (3GR4). The sphere radius was set to 10 Å. The protein skin was defined using a probe of radius σ = 3 Å and a thickness δ of 4 Å.

### The docking protocol

Potential allosteric activators were docked into the allosteric sites of the PYKs using GLIDE (Grid-based Ligand Docking with Energetics) as implemented in the Schrödinger Maestro program version 9.1 [40]–[42]. The docking solutions were analysed to identify binding poses of the ligands within the allosteric site.

Based on available data in the literature (see Table 1), the following ligands were chosen: β-D-fructose 1,6-diphosphate (FBP) (ChEBI Id: 28013), β-D-fructose 6-phosphate (F6P) (ChEBI Id: 16084), β-D-fructose 1-phosphate (F1P), β-D-glucose 6-phosphate (G6P) (ChEBI Id: 17719), β-D-galactose 6-phosphate (Gal6P) (ChEBI Id: 41076) and D-ribose 5-phosphate (R5P). The structures of the ligands were retrieved from the PubChem database [69] or generated manually with the Maestro program. The program “LigPrep”, also implemented in Maestro, was used to compute physiological protonation states for the ligands over a pH range from 5 to 9 and to energy minimize their structures.

The models of the PYKs from *Lactococcus lactis*, *Streptococcus pyogenes*, *Enterococcus faecalis*, *Lactobacillus plantarum* and S*treptococcus mutans*, and the crystal structure of the PYK from *Escherichia coli* (1PKY) were selected as targets for docking. Before docking, the PYK structures were prepared with the “Protein Preparation Wizard” from the Maestro software. Hydrogen atoms were added and the structures were checked for missing residues, atoms or secondary structure elements. Missing atoms, especially in the allosteric binding site, were reconstructed with the PRIME tool [70]. The receptor grid was centred on the allosteric site and positioned identically for all protein structures, due to their superposition. The grid box dimensions were 20 Å×20 Å×20 Å and covered the allosteric site. The initial grid spacing is predefined by the program, but was varied during the docking procedure from 2 to 0.5 Å [40]. 20 different ligand poses were computed per ligand for each protein structure. The best ligand pose was determined according to the Emodel score as suggested by Friesner and colleagues [40]. The different ligand poses were visualized and clustered manually to roughly consider entropic effects upon binding. The Emodel docking score is a composite scoring function composed of the energy-grid score, the computed binding affinity (Glide Score) and the ligand strain energy [40]. The computed binding affinities given by Glide Score alone were similar for all ligands studied and did not permit ranking corresponding to available experimental data on the allosteric effects of the compounds docked. The ranking obtained with the Emodel score however showed good consistency with experimental data available for PYK: sugar-phosphate ligand interactions. It was able to correctly assign 10 out of 11 of the experimentally observed compounds as activator or non-activator (Gal6P was wrongly predicted as favourable activator for the PYK of *Lactococcus lactis*). Therefore, the Emodel score was used to rank and compare the docked complexes of the set of six ligands with the PYKs. As a test of the applicability of the docking method to sugar-phosphate ligand-PYK complexes, we re-docked FBP into the *Saccharomyces cerevisiae* PYK structure from the crystal structure of the complex. LigPrep was used for the protonation. No further energy refinement was applied to the ligand. The best scored binding solution showed an RMSD of 0.001 Å from the crystallographically determined ligand atom positions.

### Modelling of the enzyme kinetics of *Lactococcus lactis* PYK

The kinetic model for the PYK from *Lactococcus lactis* was set up in COPASI [45] version 35.0. A reversible Hill equation was chosen as the rate law for the PYK reaction [44]:

*PEP* + *ADP* → *PYR* + *ATP*

Three different inhibition terms were initially used where i) the modifier binding was defined in a competitive formulation, ii) the modifiers bind independently of each other, and iii) a general formulation was employed that allows the modifiers to bind at separate sites at the same time. For further analysis, the competitive inhibition formulation was applied as shown in the following equation.
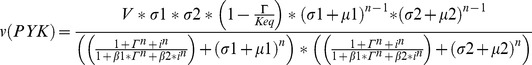

*v*(PYK) represents the reaction velocity of the PYK reaction. *V* denotes the maximum reaction velocity. *σ1* and *σ2* are the substrate concentrations, [*PEP*] and [*ADP*], divided by their binding constants, *K_m_(PEP)* and *K_m_(ADP)*, respectively. *μ1* and *μ2* represent the corresponding product terms. *Γ* and *K_eq_* are the mass action ratio term and the equilibrium constant of the PYK reaction, respectively. An equilibrium constant, *K_eq_*, of 1.2 * 10^−5^ was manually calculated for 30°C. The standard Gibbs free energy (Δ_r_G°) was estimated at the eQuilibrator webserver [71] at pH 7 and an ionic strength of 150 mM based on the compound formation energies (Δ_f_G°) as reported by Alberty [72]. *n* represents the maximum number of substrate binding sites in the enzyme complex. *a* and *i* represent the activator and inhibitor terms, respectively, similarly to the substrate formulation. *β1* and *β2* are factors that represent the interaction strength of the modifiers.

The model layout was derived from the experiments of Mason and colleagues [12] and was composed of 2 reactions: i) the PYK reaction as described previously, and ii) a sink reaction that represents the coupling enzyme (L-LDH) in the experimental set-up. The initial concentrations for the substrates [PEP], [ADP] and the product [ATP] were defined as 2 mM, and for the product [PYR] as 0 mM. The kinetic parameters were derived by parameter estimation based on the experiments of Mason and colleagues. For this purpose, experimentally determined parameters were allowed to vary +/−5-fold compared to the original values. Unknown K_m_ values for the products were estimated in an interval from 0.01 to 25 mM. The range of *β1* was defined from 1 to 250 and the interval for estimating *β2* was from 1*10^−4^ to 1. All model parameters are shown in Table S8. The algorithms, Particle Swarm [73], Random Search [74] and Simulated Annealing [75], as implemented in COPASI, were employed for parameter estimation. The model simulations were performed with the LSODA algorithm [45]. An SBML file of the kinetic model is provided in supplementary Model S1.

## Supporting Information

Dataset S1 **PYK structures and the docking solution for the best ligands.**
(ZIP)

Figure S1 **Multiple sequence alignment of the target and reference PYKs.** Multiple sequence alignment of the PYKs from *Saccharomyces cerevisiae* (P00549), *Homo sapiens* (P14618), *Escherichia coli* (P0AD61), *Enterococcus faecalis* (Q836R2), *Lactobacillus plantarum* (Q88VY2), *Streptococcus mutans* (Q8DTX7), *Lactococcus lactis* (Q07637). Residues in the active center contributing to the binding of phosphoenolpyruvate, ADP, mono- or divalent cations are marked in green [33].
(DOCX)

Figure S2 **Workflow representing the process of predicting allosteric activators for LAB PYKs.**
(TIFF)

Model S1 **SBML file for the kinetic model.**
(XML)

Table S1 **The computed phosphate interaction energies at the PYK allosteric sites.**
(DOCX)

Table S2 **Hodgkin Indices computed for the pairwise quantitative PIPSA comparison of the electrostatic potentials at the allosteric site of the PYKs.**
(DOCX)

Table S3 **Results of docking the potential activator ligands into the allosteric site of PYKs.**
(DOCX)

Table S4 **Sequence information for the modelled PYKs.**
(DOCX)

Table S5 **Assignment of residues from PYK crystal structures to the chimeric template for comparative modelling.**
(DOCX)

Table S6 **Residues excluded from comparative modelling of the PYKs.**
(DOCX)

Table S7 **Flipped Residues and unusual protonation states introduced by WHATIF upon protonation of the PYK structures.**
(DOCX)

Table S8 **Parameters of the kinetic model of the PYK from** ***Lactococcus lactis*** **.**
(DOCX)
