# Correction: Organism-Adapted Specificity of the Allosteric Regulation of Pyruvate Kinase in Lactic Acid Bacteria

**DOI:** 10.1371/journal.pcbi.1003732

**Published:** 2014-06-26

**Authors:** The PLOS Computational Biology Staff

There is an error in the nomenclature of one compound studied in this paper. The phosphate moieties of fructose 1,6-bisphosphate (FBP) were incorrectly labelled with the 1’-phosphate being labelled 6’ and the 6’-phosphate being labelled 1’. The corrected figure legend and sentences are:

In Results/Discussion, Section “PYKs show a conserved phosphate binding site in the allosteric activator binding site”, paragraph 1, 8th sentence: The phosphate interaction sites that coincide with the 6′-phosphate and the 1′-phosphate moieties of FBP are labelled 1′Pibs and 6′Pibs, respectively.

In Results/Discussion, Section “Lactic acid bacteria PYKs lack a second phosphate binding site in the activator binding site”, paragraph 1, 2nd sentence: In the yeast and the human M2-type PYKs, the side chains of tryptophan (W452 in 1A3W and W482 in 3GR4) and arginine (R459 in 1A3W and R489 in 3GR4) residues are computed to interact with the 1′-phosphate moiety of FBP (see Figure 1).

**Figure 1:**
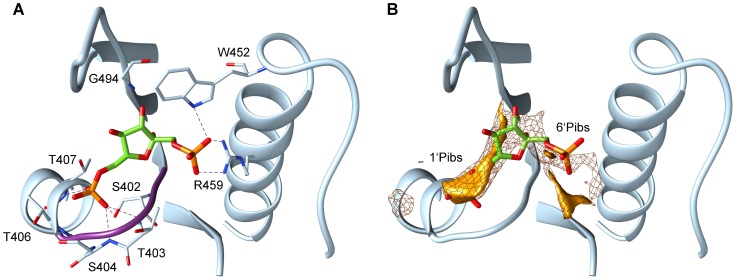
Allosteric binding site of PYK with the activator FBP bound. Residues V400 to V411, K446 to F470 and S481 to Q496 of the crystallographically resolved Saccharomyces cerevisiae PYK (PDB id: 1A3W, chain A) are shown in cartoon representation. Panel A shows possible hydrogen bonds between the FBP phosphate groups and the residues of PYK within a distance of 3 Å (all in stick representation). The structural P-loop motif (STSG) is coloured in purple. Panel B illustrates the phosphate interaction sites computed with the GRID program. The binding site of the 6′-phosphate moiety of FBP in the allosteric site is referred to as 1′Pibs and that of the 1′-phosphate moiety of FBP as 6′Pibs. The interaction energy is displayed at isocontours of −10 kcal/mol (mesh surface) and −13.5 kcal/mol (solid surface). Only the phosphate interaction sites that are located within 6 Å around FBP are shown.
