# Supplementary material for: Organism-Adapted Specificity of the Allosteric Regulation of Pyruvate Kinase in Lactic Acid Bacteria
Source: PLoS Comput Biol. 2013 Jul 25;9(7):e1003159. doi: 10.1371/journal.pcbi.1003159 (PMC3738050; doi:10.1371/journal.pcbi.1003159)
Supplement: Figure S1 — Multiple sequence alignment of the target and reference PYKs. Multiple sequence alignment of the PYKs from Saccharomyces cerevisiae (P00549), Homo sapiens (P14618), Escherichia coli (P0AD61), Enterococcus faecalis (Q836R2), Lactobacillus plantarum (Q88VY2), Streptococcus mutans (Q8DTX7), Lactococcus lactis (Q07637). Residues in the active center contributing to the binding of phosphoenolpyruvate, ADP, mono- or divalent cations are marked in green [33]. (DOCX) [file pcbi.1003159.s002.docx]

Supplementary Figure S1:

sp|P00549|KPYK1_YEAST MSR-----------------------LERLTSLNVVAG-SDLRRTSIIGT 26

sp|P14618|KPYM_HUMAN MSKPHSEAGTAFIQTQQLHAAMADTFLEHMCRLDIDSPPITARNTGIICT 50

sp|P0AD61|KPYK1_ECOLI -----------------------------------------MKKTKIVCT 9

tr|Q836R2|Q836R2_ENTFA -----------------------------------------MKKTKIVCT 9

tr|Q88VY2|Q88VY2_LACPL -----------------------------------------MKKTKIVST 9

tr|B5XLV5|B5XLV5_STRPZ ----------------------------------------MNKRVKIVAT 10

tr|Q8DTX7|Q8DTX7_STRMU ----------------------------------------MNKRVKIVAT 10

sp|Q07637|KPYK_LACLA ----------------------------------------MNKRVKIVST 10

:.. *: *

sp|P00549|KPYK1_YEAST IGPKTNN---------------------PETLVALRKAGLNIVRMNFSHG 55

sp|P14618|KPYM_HUMAN IGPASRS---------------------VETLKEMIKSGMNVARLNFSHG 79

sp|P0AD61|KPYK1_ECOLI IGPKTES---------------------EEMLAKMLDAGMNVMRLNFSHG 38

tr|Q836R2|Q836R2_ENTFA IGPASES---------------------VDMLVNLINAGMNVCRLNFSHG 38

tr|Q88VY2|Q88VY2_LACPL LGPASTD---------------------TDTIVKLIEAGANIFRFNFSHG 38

tr|B5XLV5|B5XLV5_STRPZ LGPAVEIRGGKKYGEDGYWAGQLDVEESAKKIAELIEAGANVFRFNFSHG 60

tr|Q8DTX7|Q8DTX7_STRMU LGPAVEIRGGKKFGEDGYWGEKLDVEASAAKIAELITEGANVFRFNFSHG 60

sp|Q07637|KPYK_LACLA LGPAVEIRGGKKFGESGYWGESLDVEASAKNIAALIEEGANVFRFNFSHG 60

:** : : * *: *:*****

sp|P00549|KPYK1_YEAST SYEYHKSVIDNARKSEELYPG-----RPLAIALDTKGPEIRTGTTTN--D 98

sp|P14618|KPYM_HUMAN THEYHAETIKNVRTATESFASDPILYRPVAVALDTKGPEIRTGLIKGSGT 129

sp|P0AD61|KPYK1_ECOLI DYAEHGQRIQNLRN-VMSKTG-----KTAAILLDTKGPEIRTMKLEG--G 80

tr|Q836R2|Q836R2_ENTFA DYEEHGARIKNIREAVKITGK------RVAILLDTKGPEIRT-NDMENGA 81

tr|Q88VY2|Q88VY2_LACPL DHEEHLDRLNKVHEAEKITGK------TVGIMLDTKGAEIRT-TVQANGK 81

tr|B5XLV5|B5XLV5_STRPZ DHKEQGDRMATVRLAEEIARQ------KVGFLLDTKGPEMRT-ELFADDA 103

tr|Q8DTX7|Q8DTX7_STRMU DHAEQGERMATVRRAEELARQ------KVGFLLDTKGPEMRT-ELFADGV 103

sp|Q07637|KPYK_LACLA DHPEQGARMATVHRAEEIAGH------KVGFLLDTKGPEMRT-ELFADGA 103

: : : . : .. *****.*:**

sp|P00549|KPYK1_YEAST VDYPIPPNHEMIFTTDDKYAKACDDKIMYVD-YKNITKVISAGRIIYVDD 147

sp|P14618|KPYM_HUMAN AEVELKKGATLKITLDNAYMEKCDENILWLD-YKNICKVVEVGSKIYVDD 178

sp|P0AD61|KPYK1_ECOLI NDVSLKAGQTFTFTTDKSVIG--NSEMVAVT-YEGFTTDLSVGNTVLVDD 127

tr|Q836R2|Q836R2_ENTFA --ITMKIGDSVRISMTEVLG-TNEKFSITYP---ELINDVNVGSHILLDD 125

tr|Q88VY2|Q88VY2_LACPL --SEYKIGDKVRITMDDSLDTTHDKIAVTYK---NLYDDVHVGGHVLFDD 126

tr|B5XLV5|B5XLV5_STRPZ KEFSYVTGEKIRVATTQGIQSTRDVIALNVAGSLDIYDEVEVGHTILIDD 153

tr|Q8DTX7|Q8DTX7_STRMU KEYEYKTGDKLRIATKQGIESTKDVIALNVAGGLDIYDDVAVGQTILIDD 153

sp|Q07637|KPYK_LACLA DAISVVTGDKFRVATKQGLKSTPELIALNVAGGLDIFDDVEIGQTILIDD 153

. . .: . : : : : * : .**

sp|P00549|KPYK1_YEAST GVLSFQVLEVVDDK-TLKVKALNAGKICSHKGVNLPGTDVDLPALSEKDK 196

sp|P14618|KPYM_HUMAN GLISLQVKQKGAD--FLVTEVENGGSLGSKKGVNLPGAAVDLPAVSEKDI 226

sp|P0AD61|KPYK1_ECOLI GLIGMEVTAIEGN--KVICKVLNNGDLGENKGVNLPGVSIALPALAEKDK 175

tr|Q836R2|Q836R2_ENTFA GLIDLEVTDIDRDANEIVTVVKNEGVLKNKKGVNVPGVSVNLPGITEKDA 175

tr|Q88VY2|Q88VY2_LACPL GLLDMKIDEKDEANRELVTTVQNAGVLGSRKGVNAPGVSINLPGITEKDS 176

tr|B5XLV5|B5XLV5_STRPZ GKLGLKVIDKDIATRQFIVEVENDGIIAKQKGVNIPNTKIPFPALAERDN 203

tr|Q8DTX7|Q8DTX7_STRMU GKLGLTVTAKDITTREFEVTVENDGIIAKQKGVNIPNTKIPFPALAERDN 203

sp|Q07637|KPYK_LACLA GKLGLSLTGKDAATREFEVEAQNDGVIGKQKGVNIPNTKIPFPALAERDD 203

* :.: : . . * * : ..**** *.. : :*.::*:*

sp|P00549|KPYK1_YEAST EDLRFGVKN--GVHMVFASFIRTANDVLTIREVLGEQG-KDVKIIVKIEN 243

sp|P14618|KPYM_HUMAN QDLKFGVEQ--DVDMVFASFIRKASDVHEVRKVLGEKG-KNIKIISKIEN 273

sp|P0AD61|KPYK1_ECOLI QDLIFGCEQ--GVDFVAASFIRKRSDVIEIREHLKAHGGENIHIISKIEN 223

tr|Q836R2|Q836R2_ENTFA NDIRFGIGQ--GIDFIAASFVRRASDVLEITKILEEENATHIQIIPKIEN 223

tr|Q88VY2|Q88VY2_LACPL SDIRFGLDH--EINYIAASFVRKPQDVLDIRELLEEKHMEHVQIFPKIES 224

tr|B5XLV5|B5XLV5_STRPZ ADIRFGLEQ--GLNFIAISFVRTAKDVEEVREICRETGNDHVQLFAKIEN 251

tr|Q8DTX7|Q8DTX7_STRMU ADIRFGLEQ--GLNFIAISFVRTAKDVNEVRQICKETGNEHVKLFAKIEN 251

sp|Q07637|KPYK_LACLA ADIRFGLSQPGGINFIAISFVRTANDVKEVRRICEETGNPHVQLLAKIEN 253

*: ** : :. : **:* .** : . .:::: ***.

sp|P00549|KPYK1_YEAST QQGVNNFDEILKVTDGVMVARGDLGIEIPAPEVLAVQKKLIAKSNLAGKP 293

sp|P14618|KPYM_HUMAN HEGVRRFDEILEASDGIMVARGDLGIEIPAEKVFLAQKMMIGRCNRAGKP 323

sp|P0AD61|KPYK1_ECOLI QEGLNNFDEILEASDGIMVARGDLGVEIPVEEVIFAQKMMIEKCIRARKV 273

tr|Q836R2|Q836R2_ENTFA QEGIDNIDEILKVSDGLMVARGDMGVEIPTEDVPVVQKALIKKCNALGKP 273

tr|Q88VY2|Q88VY2_LACPL QEGIDNADEILKVCDGLMVARGDMGVEIPAENVPLVQKSLIKKCNALGMP 274

tr|B5XLV5|B5XLV5_STRPZ QQGIDNLDEIIEAADGIMIARGDMGIEVPFEMVPVFQKMIITKVNAAGKA 301

tr|Q8DTX7|Q8DTX7_STRMU QQGIDNIDEIIDAADGIMIARGDMGIEVPFEMVPVYQKMIITKVNAAGKS 301

sp|Q07637|KPYK_LACLA QQGIENLDEIIEAADGIMIARGDMGIEVPFEMVPVYQKLIISKVNKAGKI 303

::*: . ***:.. **:*:****:*:*:* * ** :* :

sp|P00549|KPYK1_YEAST VICATQMLESMTYNPRPTRAEVSDVGNAILDGADCVMLSGETAKGNYPIN 343

sp|P14618|KPYM_HUMAN VICATQMLESMIKKPRPTRAEGSDVANAVLDGADCIMLSGETAKGDYPLE 373

sp|P0AD61|KPYK1_ECOLI VITATQMLDSMIKNPRPTRAEAGDVANAILDGTDAVMLSGESAKGKYPLE 323

tr|Q836R2|Q836R2_ENTFA VITATQMLDSMQRNPRPTRAEANDVANAIYDGTDAVMLSGETAAGDYPLE 323

tr|Q88VY2|Q88VY2_LACPL VITATQMLDSMQENPRPTRAEASDVANAVFDGTDATMLSGESANGLYPVE 324

tr|B5XLV5|B5XLV5_STRPZ VITATNMLETMTEKPRATRSEVSDVFNAVIDGTDATMLSGESANGKYPVE 351

tr|Q8DTX7|Q8DTX7_STRMU AITATNMLETMTDKPRATRSEVSDVFNAVIDGTDATMLSGESANGKYPVE 351

sp|Q07637|KPYK_LACLA VVTATNMLESMTYNPRATRSEISDVFNAVIDGTDATMLSGESANGKYPRE 353

.: **:**::* :**.**:* .** **: **:*. *****:* * ** :

sp|P00549|KPYK1_YEAST AVTTMAETAVIAEQAIAY-LPNYDDMRNCTPKPTSTTETVAASAVAAVFE 392

sp|P14618|KPYM_HUMAN AVRMQHLIAREAEAAIYH-LQLFEELRRLAPITSDPTEATAVGAVEASFK 422

sp|P0AD61|KPYK1_ECOLI AVSIMATICERTDRVMNSRLEFNNDNRKLR-----ITEAVCRGAVETAEK 368

tr|Q836R2|Q836R2_ENTFA AVQTMARIAVRTEETLVN----QDSFALKLYSKTDMTEAIGQSVGHTARN 369

tr|Q88VY2|Q88VY2_LACPL SVAMMAKIDEKAENTLAE----NGTLQLNRFDKTSVTETIGIAIARAAKN 370

tr|B5XLV5|B5XLV5_STRPZ SVRTMATIDRNAQTLLNE----YGRLDSSAFPRTNKTDVIASAVKDATHS 397

tr|Q8DTX7|Q8DTX7_STRMU AVRTMATIDKNAQTLLNE----YGRLNSDNLPRTNKTEVVASAVKDATKS 397

sp|Q07637|KPYK_LACLA SVRTMATVNKNAQTMLKE----YGRLHPERYDKSTVTEVVAASVKNAAEA 399

:* :: : *:. . :

sp|P00549|KPYK1_YEAST QKAKAIIVLSTSGTTPRLVSKYRPNCPIILVTRCPRAARFSHLYRGVFPF 442

sp|P14618|KPYM_HUMAN CCSGAIIVLTKSGRSAHQVARYRPRAPIIAVTRNPQTARQAHLYRGIFPV 472

sp|P0AD61|KPYK1_ECOLI LDAPLIVVATQGGKSARAVRKYFPDATILALTTNEKTAHQLVLSKGVVPQ 418

tr|Q836R2|Q836R2_ENTFA LGIQTIVAATESGHTARMISKYRPKAHIVAITFSEQKARSLSLSWGVYAT 419

tr|Q88VY2|Q88VY2_LACPL LNIKTIVAATESGYTAKMISKYRPNADILAITFDERTQRGLMVNWGVQPI 420

tr|B5XLV5|B5XLV5_STRPZ MDIKLVVTITETGNTARAISKFRPDADILAVTFDEKVQRALMINWGVIPV 447

tr|Q8DTX7|Q8DTX7_STRMU MDIKLVVTITESGNTARLISKYRPDADILALTFDEKVQKSLMINWGVIPI 447

sp|Q07637|KPYK_LACLA MDIKLIVALTESGNTARLISKHRPNADILAITFDEKVERGLMINWGVIPT 449

::. : * :.: : :. * . *: :* : : : *: .

sp|P00549|KPYK1_YEAST VFEKEPVSDWTDDVEARINFGIEKAKEFGILKKGDTYVSIQGFKAGA-GH 491

sp|P14618|KPYM_HUMAN LCKDPVQEAWAEDVDLRVNFAMNVGKARGFFKKGDVVIVLTGWRPGS-GF 521

sp|P0AD61|KPYK1_ECOLI LVKEITS---TDDFYR---LGKELALQSGLAHKGDVVVMVSGALVPS-GT 461

tr|Q836R2|Q836R2_ENTFA VADKPSS---TDEMFN---LASKVSQEEGYASEGDLIIITAGVPVGEKGT 463

tr|Q88VY2|Q88VY2_LACPL VAEKPET---TDDMFD---LAASKAVELGFAKEGDLILITAGVPVGERGT 464

tr|B5XLV5|B5XLV5_STRPZ LAEKPAS---TDDMFE---VAERVAVEAGLVQSGDNIVIVAGVPVGT-GG 490

tr|Q8DTX7|Q8DTX7_STRMU LTEKPAS---TDDMFE---IAEKAALKSGLVESGDNIVIVAGVPVGS-GG 490

sp|Q07637|KPYK_LACLA MTEKPSS---TDDMFE---VAEKVALASGLVESGDNIIIVAGVPVGT-GR 492

: .. :::. .. . * .** : * *

sp|P00549|KPYK1_YEAST SNTLQVSTV----------------------------------------- 500

sp|P14618|KPYM_HUMAN TNTMRVVPVP---------------------------------------- 531

sp|P0AD61|KPYK1_ECOLI TNTASVHVL----------------------------------------- 470

tr|Q836R2|Q836R2_ENTFA TNLMKIQMIGSKLVQGQGVGEEAIIAKAVVAATAEEAVAKATEGAILVTK 513

tr|Q88VY2|Q88VY2_LACPL TNIMKIQLIGSKLADGQGVGDETVIGKAVIATSAQEAIDKAVEGGVLVTK 514

tr|B5XLV5|B5XLV5_STRPZ TNTMRVRTVK---------------------------------------- 500

tr|Q8DTX7|Q8DTX7_STRMU TNTMRVRTVQ---------------------------------------- 500

sp|Q07637|KPYK_LACLA TNTMRIRTVK---------------------------------------- 502

:* : :

sp|P00549|KPYK1_YEAST --------------------------------------------------

sp|P14618|KPYM_HUMAN --------------------------------------------------

sp|P0AD61|KPYK1_ECOLI --------------------------------------------------

tr|Q836R2|Q836R2_ENTFA TTDKEYMPAIEKASALVVEEGGLTSHAAVVAIAQNIPVIVGAADATSLIN 563

tr|Q88VY2|Q88VY2_LACPL TTDKDYLPAIEKSSALVVENGGLTSHAAVVGISMGIPVIVGVKDATSVIA 564

tr|B5XLV5|B5XLV5_STRPZ --------------------------------------------------

tr|Q8DTX7|Q8DTX7_STRMU --------------------------------------------------

sp|Q07637|KPYK_LACLA --------------------------------------------------

sp|P00549|KPYK1_YEAST ----------------------

sp|P14618|KPYM_HUMAN ----------------------

sp|P0AD61|KPYK1_ECOLI ----------------------

tr|Q836R2|Q836R2_ENTFA NDEVITVDPRRGIVYRGATTAI 585

tr|Q88VY2|Q88VY2_LACPL DGQLITVDSRRGLVYRGASNAL 586

tr|B5XLV5|B5XLV5_STRPZ ----------------------

tr|Q8DTX7|Q8DTX7_STRMU ----------------------

sp|Q07637|KPYK_LACLA ----------------------
