# Supplementary figures and images for: Organism-Adapted Specificity of the Allosteric Regulation of Pyruvate Kinase in Lactic Acid Bacteria
Source: PLoS Comput Biol. 2013 Jul 25;9(7):e1003159. doi: 10.1371/journal.pcbi.1003159 (PMC3738050; doi:10.1371/journal.pcbi.1003159)

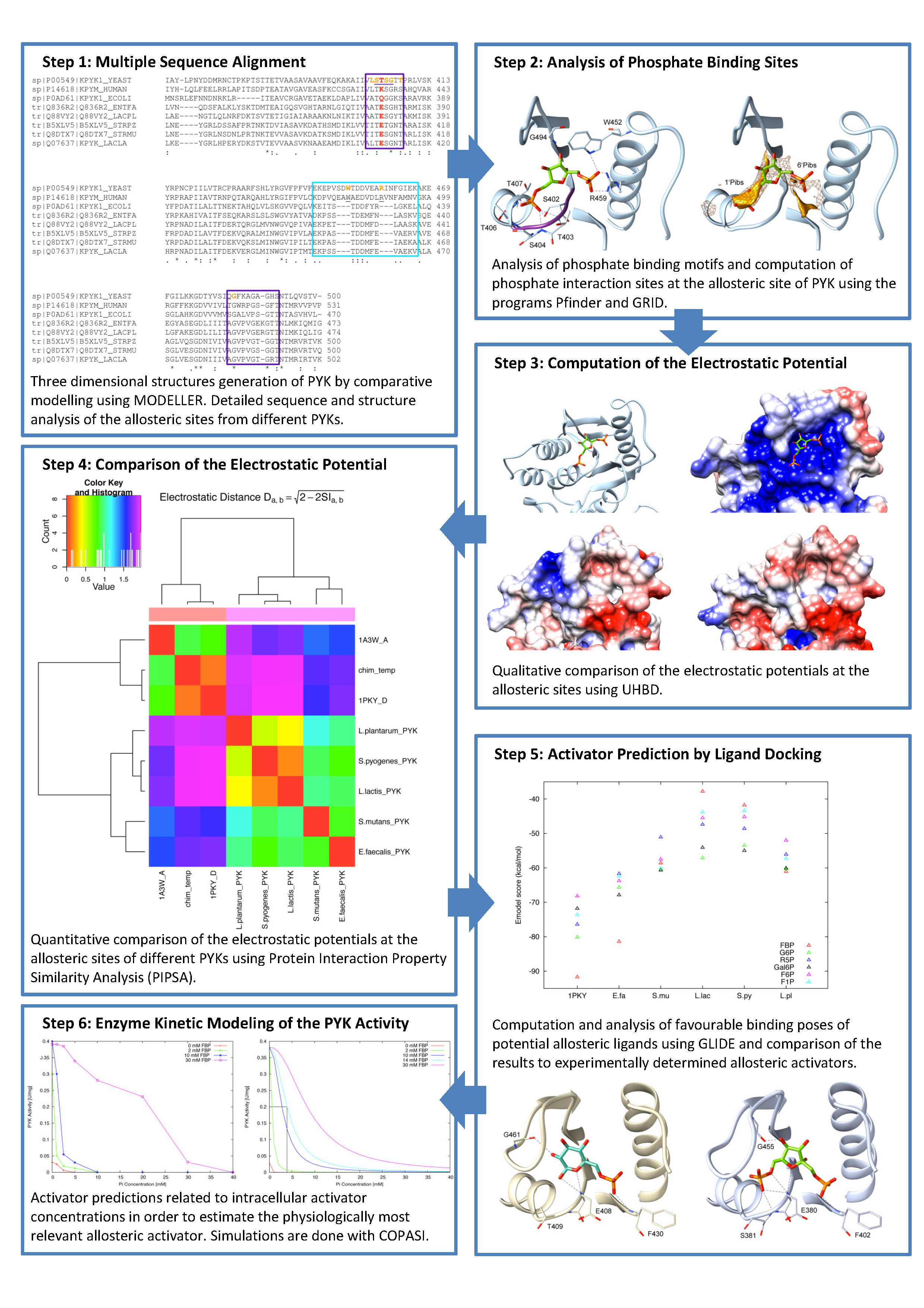

Supplement: Figure S2 — Workflow representing the process of predicting allosteric activators for LAB PYKs. (TIFF) [file pcbi.1003159.s003.tiff]
