# Supplementary material for: Organism-Adapted Specificity of the Allosteric Regulation of Pyruvate Kinase in Lactic Acid Bacteria
Source: PLoS Comput Biol. 2013 Jul 25;9(7):e1003159. doi: 10.1371/journal.pcbi.1003159 (PMC3738050; doi:10.1371/journal.pcbi.1003159)
Supplement: Table S1 — The computed phosphate interaction energies at the PYK allosteric sites. (DOCX) [file pcbi.1003159.s005.docx]

Supplementary Table S1

| **The computed phosphate interaction energies at the PYK allosteric sites** | | |
| --- | --- | --- |
| **PYK origin^*^** | **Most favorable interaction energy [kcal/mol]** | |
|  | **1’Pibs** | **6’Pibs** |
| *Saccharomyces cerevisiae* (1A3W) | -26 | -14 |
| *Homo Sapiens* (3GR4) | -24 | -17 |
| *Escherichia coli* (1PKY) | -22 | -10 |
|  |  |  |
| *Lactococcus lactis* | -11 | -4 |
| *Streptococcus mutans* | -13 | -7 |
| *Streptococcus pyogenes* | -10 | -5 |
| *Enterococcus faecalis* | -9 | -3 |
| *Lactobacillus plantarum* | -14 | -7 |

* PDB file identifiers are given in brackets for the three crystal structures analysed. The structures for all the LAB PYKs were made by comparative modeling.
