# Supplementary material for: Organism-Adapted Specificity of the Allosteric Regulation of Pyruvate Kinase in Lactic Acid Bacteria
Source: PLoS Comput Biol. 2013 Jul 25;9(7):e1003159. doi: 10.1371/journal.pcbi.1003159 (PMC3738050; doi:10.1371/journal.pcbi.1003159)
Supplement: Table S2 — Hodgkin Indices computed for the pairwise quantitative PIPSA comparison of the electrostatic potentials at the allosteric site of the PYKs. (DOCX) [file pcbi.1003159.s006.docx]

Supplementary Table S2:

| **Hodgkin indices computed for the pairwise quantitative PIPSA comparison of the electrostatic potentials at the allosteric site of the PYKs^*^** | | | | | | | | |
| --- | --- | --- | --- | --- | --- | --- | --- | --- |
|  | Chimeric template | 1PKY_D | *L. plantarum* PYK | *S. pyogenes* PYK | *S. mutans* PYK | *E. faecalis* PYK | 1A3W_A | *L. lactis* PYK |
| Chimeric template | 1.000 | 0.992 | -0.787 | -0.876 | -0.354 | -0.419 | 0.616 | -0.882 |
| 1PYK_D | 0.992 | 1.000 | -0.756 | -0.903 | -0.274 | -0.482 | 0.671 | -0.898 |
| *L. plantarum* PYK | -0.787 | -0.756 | 1.000 | 0.875 | 0.369 | 0.521 | -0.685 | 0.928 |
| *S. pyogenes* PYK | -0.876 | -0.903 | 0.875 | 1.000 | 0.593 | 0.681 | -0.420 | 0.987 |
| *S. mutans* PYK | -0.354 | -0.274 | 0.369 | 0.593 | 1.000 | 0.651 | -0.043 | 0.524 |
| *E. faecalis* PYK | -0.419 | -0.482 | 0.521 | 0.681 | 0.651 | 1.000 | -0.134 | 0.584 |
| 1A3W_A | 0.616 | 0.671 | -0.685 | -0.420 | -0.043 | -0.134 | 1.000 | -0.491 |
| *L. lactis* PYK | -0.882 | -0.898 | 0.928 | 0.987 | 0.524 | 0.584 | -0.491 | 1.000 |

* Values of the pairwise Hodgkin indices vary between 1.0, indicating identical electrostatic potentials in the region compared, and -1.0 indicating opposite potentials.
