# Supplementary material for: Organism-Adapted Specificity of the Allosteric Regulation of Pyruvate Kinase in Lactic Acid Bacteria
Source: PLoS Comput Biol. 2013 Jul 25;9(7):e1003159. doi: 10.1371/journal.pcbi.1003159 (PMC3738050; doi:10.1371/journal.pcbi.1003159)
Supplement: Table S3 — Results of docking the potential activator ligands into the allosteric site of PYKs. (DOCX) [file pcbi.1003159.s007.docx]

| **Results of docking the potential activator ligands into the allosteric site of *Escherichia coli* PYK (1PKY)** | | | | | | | |
| --- | --- | --- | --- | --- | --- | --- | --- |
| **Ligands** | **most favorable E_model_ score^*^**  **[kcal/mol]** | **Total number of binding poses** | **Probability of binding pose [%]^$^** | **E_model_  score per binding pose^§^ [kcal/mol]** | **Orientation of the P_i_-moiety^#^** | **Number of solutions**  **not clustered in a binding pose** | **Number of solutions not docked in the allosteric site** |
| FBP | -91.8 | 4 | 30 | -86.8 | - | 2 | 1 |
|  |  |  | 25 | -88.1 | - |  |  |
|  |  |  | 10 | -91.7 | - |  |  |
|  |  |  | 10 | -86.3 | - |  |  |
|  |  |  |  |  |  |  |  |
| G6P | -80.2 | 4 | 60 | -80.2 | 1’Pi | - | 1 |
|  |  |  | 10 | -63.9 | 1’Pi |  |  |
|  |  |  | 10 | -74.8 | 1’Pi |  |  |
|  |  |  | 10 | -70.9 | 6’Pi |  |  |
|  |  |  |  |  |  |  |  |
| R5P | -76.4 | 2 | 25 | -76.4 | 1’Pi | 3 | 3 |
|  |  |  | 45 | -73.0 | 1’Pi |  |  |
|  |  |  |  |  |  |  |  |
| F1P | -73.6 | 2 | 15 | -71.3 | 1’Pi | 8 | 2 |
|  |  |  | 35 | -73.6 | 1’Pi |  |  |
|  |  |  |  |  |  |  |  |
| Gal6P | -71.8 | 3 | 35 | -70.0 | 1’Pi | 2 | 4 |
|  |  |  | 5 | -67.1 | 1’Pi |  |  |
|  |  |  | 20 | -71.8 | 1’Pi |  |  |
|  |  |  |  |  |  |  |  |
| F6P | -68.2 | 3 | 50 | -66.8 | 1’Pi | 3 | 2 |
|  |  |  | 10 | -68.2 | 1’Pi |  |  |
|  |  |  | 15 | -61. 8 | 1’Pi |  |  |
|  |  |  |  |  |  |  |  |
| Gal1P | -47.7 | 2 | 10 | -43.6 | 1’Pi | 2 | 3 |
|  |  |  | 75 | -47.7 | 1’Pi |  |  |

Supplementary Table S3:

^*^ Most favourable Emodel docking score obtained amongst the 20 docking solutions for each ligand

^$^ Probability (number of docking solutions per cluster/ total number of docking solutions obtained in the allosteric site) that a ligand docking solution fits the corresponding binding pose cluster

^§^ Most negative (favourable) binding pose score within each binding pose cluster

^#^ Orientation of the phosphate moiety towards the 1’Pibs or the 6’Pibs site.

| **Results of docking the potential activator ligands into the allosteric site of *Streptococcus mutans* PYK** | | | | | | | |
| --- | --- | --- | --- | --- | --- | --- | --- |
| **Ligands** | **most favorable E_model_ score^*^**  **[kcal/mol]** | **Total number of binding poses** | **Probability of binding pose [%]^$^** | **E_model_  score per binding pose^§^ [kcal/mol]** | **Orientation of the P_i_-moiety^#^** | **Number of solutions**  **not clustered in a binding pose** | **Number of solutions not docked in the allosteric site** |
| Gal6P | -60.7 | 4 | 40 | -60.7 | 6’ P_i_ | - | - |
|  |  |  | 5 | -58.8 | 6’ P_i_ |  |  |
|  |  |  | 45 | -55.8 | 6’ P_i_ |  |  |
|  |  |  | 10 | -43.4 | 6’ P_i_ |  |  |
|  |  |  |  |  |  |  |  |
| G6P | -60.5 | 5 | 30 | -59.2 | 6’ P_i_ | - | 1 |
|  |  |  | 10 | -55.2 | 6’ P_i_ |  |  |
|  |  |  | 40 | -60.5 | 6’ P_i_ |  |  |
|  |  |  | 10 | -48.2 | 1’ P_i_ |  |  |
|  |  |  | 5 | -45.3 |  |  |  |
|  |  |  |  |  |  |  |  |
| F1P | -60.1 | 2 | 30 | -60.1 | 6’ P_i_ | 7 | 1 |
|  |  |  | 30 | -52.5 | 6’ P_i_ |  |  |
|  |  |  |  |  |  |  |  |
| FBP | -58.6 | 3 | 10 | -58.6 | - | 5 | 6 |
|  |  |  | 20 | -53.4 |  |  |  |
|  |  |  | 15 | -49.4 |  |  |  |
|  |  |  |  |  |  |  |  |
| F6P | -57.5 | 1 | 65 | -57.5 | 6’ P_i_ | 3 | 3 |
|  |  |  |  |  |  |  |  |
| R5P | -51.1 | 2 | 65 | -51.1 | 6’ P_i_ | - | 1 |
|  |  |  | 30 | -50.5 | 6’ P_i_ |  |  |
|  |  |  |  |  |  |  |  |
| Gal1P | -36.8 | 3 | 30 | -35.7 | 1’ P_i_ | - | 1 |
|  |  |  | 25 | -34.0 | 1’ P_i_ |  |  |
|  |  |  | 40 | -36.9 | 6’ P_i_ |  |  |

| **Results of docking the potential activator ligands into the allosteric site of *Lactococcus lactis* PYK** | | | | | | | |
| --- | --- | --- | --- | --- | --- | --- | --- |
| **Ligands** | **most favorable E_model_ score^*^**  **[kcal/mol]** | **Total number of binding poses** | **Probability of binding pose [%]^$^** | **E_model_  score per binding pose^§^ [kcal/mol]** | **Orientation of the P_i_-moiety^#^** | **Number of solutions**  **not clustered in a binding pose** | **Number of solutions not docked in the allosteric site** |
| G6P | -57.1 | 3 | 30 | -57.1 | 6’ Pi | - | 2 |
|  |  |  | 40 | -49.5 | 6’ Pi |  |  |
|  |  |  | 20 | -40.4 | 1’ Pi |  |  |
|  |  |  |  |  |  |  |  |
| Gal6P | -54.1 | 3 | 70 | -54.1 | 6’ Pi | - | 1 |
|  |  |  | 10 | -50.2 | 6’ Pi |  |  |
|  |  |  | 15 | -38.6 | 1’ Pi |  |  |
|  |  |  |  |  |  |  |  |
| R5P | -47.4 | 2 | 70 | -47.4 | 6’ Pi | - | 2 |
|  |  |  | 20 | -46.5 | 6’ Pi |  |  |
|  |  |  |  |  |  |  |  |
| F6P | -45.5 | 2 | 35 | -45.5 | 6’ Pi | - | 5 |
|  |  |  | 45 | -35.5 | 6’ Pi |  |  |
|  |  |  |  |  |  |  |  |
| F1P | -43.8 | 2 | 30 | -43.8 | 6’ Pi | 5 | 3 |
|  |  |  | 30 | -41.9 | 6’ Pi |  |  |
|  |  |  |  |  |  |  |  |
| FBP | -37.8 | 3 | 40 | -37.8 | - | - | 6 |
|  |  |  | 20 | -33.3 |  |  |  |
|  |  |  | 10 | -30.0 |  |  |  |

| **Results of docking the potential activator ligands into the allosteric site of *Streptococcus pyogenes* PYK** | | | | | | | |
| --- | --- | --- | --- | --- | --- | --- | --- |
| **Ligands** | **most favorable E_model_ score^*^**  **[kcal/mol]** | **Total number of binding poses** | **Probability of binding pose [%]^$^** | **E_model_  score per binding pose^§^ [kcal/mol]** | **Orientation of the P_i_-moiety^#^** | **Number of solutions**  **not clustered in a binding pose** | **Number of solutions not docked in the allosteric site** |
| Gal6P | -55.0 | 2 | 85 | -55.0 | 6’ P_i_ | - | 1 |
|  |  |  | 10 | -51.1 | 6’ P_i_ |  |  |
|  |  |  |  |  |  |  |  |
| G6P | -53.5 | 3 | 20 | -49.5 | 6’ P_i_ | - | 2 |
|  |  |  | 35 | -53.5 | 6’ P_i_ |  |  |
|  |  |  | 35 | -49.6 | 6’ P_i_ |  |  |
|  |  |  |  |  |  |  |  |
| R5P | -48.7 | 2 | 20 | -46.5 | 6’ P_i_ | - | 1 |
|  |  |  | 75 | -48.7 | 6’ P_i_ |  |  |
|  |  |  |  |  |  |  |  |
| F6P | -45.2 | 4 | 20 | -45.0 | 6’ P_i_ | - | 1 |
|  |  |  | 15 | -42.4 | 6’ P_i_ |  |  |
|  |  |  | 35 | -45.2 | 6’ P_i_ |  |  |
|  |  |  | 25 | -44.0 | 6’ P_i_ |  |  |
|  |  |  |  |  |  |  |  |
| F1P | -43.4 | 3 | 20 | -43.4 | 6’ P_i_ | - | 1 |
|  |  |  | 50 | -42.1 | 6’ P_i_ |  |  |
|  |  |  | 25 | -43.1 | 6’ P_i_ |  |  |
|  |  |  |  |  |  |  |  |
| FBP | -41.8 | 3 | 35 | -41.8 | - | - | 7 |
|  |  |  | 20 | -37.6 |  |  |  |
|  |  |  | 10 | -34.7 |  |  |  |
|  |  |  |  |  |  |  |  |
| Gal1P | -32.6 | 3 | 40 | -31.7 | 6’ P_i_ | - | 2 |
|  |  |  | 15 | -31.1 | 6’ P_i_ |  |  |
|  |  |  | 35 | -32.6 | 1’ P_i_ |  |  |

| **Results of docking the potential activator ligands into the allosteric site of *Lactobacillus plantarum* PYK** | | | | | | | |
| --- | --- | --- | --- | --- | --- | --- | --- |
| **Ligands** | **most favorable E_model_ score^*^**  **[kcal/mol]** | **Total number of binding poses** | **Probability of binding pose [%]^$^** | **E_model_  score per binding pose^§^ [kcal/mol]** | **Orientation of the P_i_-moiety^#^** | **Number of solutions**  **not clustered in a binding pose** | **Number of solutions not docked in the allosteric site** |
| FBP | -61.1 | 5 | 10 | -59.4 | - | - | 3 |
|  |  |  | 10 | -56.5 |  |  |  |
|  |  |  | 40 | -61.1 |  |  |  |
|  |  |  | 15 | -51.5 |  |  |  |
|  |  |  | 10 | -48.7 |  |  |  |
|  |  |  |  |  |  |  |  |
| G6P | -60.4 | 5 | 35 | -60.4 | 6’ Pi | - | 3 |
|  |  |  | 10 | -57.1 | 6’ Pi |  |  |
|  |  |  | 10 | -45.9 | 6’ Pi |  |  |
|  |  |  | 20 | -49.7 | 1’ Pi |  |  |
|  |  |  | 10 | -46.3 | 1’ Pi |  |  |
|  |  |  |  |  |  |  |  |
| Gal6P | -60.1 | 6 | 20 | -57.9 | 6’ Pi | - | - |
|  |  |  | 15 | -53.0 | 6’ Pi |  |  |
|  |  |  | 10 | -55.7 | 6’ Pi |  |  |
|  |  |  | 30 | -54.2 | 1’ Pi |  |  |
|  |  |  | 15 | -60.1 | 1’ Pi |  |  |
|  |  |  | 10 | -43.9 | 1’ Pi |  |  |
|  |  |  |  |  |  |  |  |
| F1P | -57.3 | 4 | 30 | -57.3 | 6’ Pi | - | - |
|  |  |  | 15 | -56.6 | 6’ Pi |  |  |
|  |  |  | 45 | -50.6 | 1’ Pi |  |  |
|  |  |  | 10 | -44.2 | 1’ Pi |  |  |
|  |  |  |  |  |  |  |  |
| R5P | -56.1 | 2 | 65 | -56.1 | 6’ Pi | - | 4 |
|  |  |  | 15 | -50.5 | 1’ Pi |  |  |
|  |  |  |  |  |  |  |  |
| F6P | -52.0 | 3 | 75 | -52.0 | 6’ Pi | - | - |
|  |  |  | 10 | -49.9 | 1’ Pi |  |  |
|  |  |  | 15 | -44.3 | 1’ Pi |  |  |
|  |  |  |  |  |  |  |  |
| Gal1P | -39.8 | 4 | 45 | -38.1 | 1’ Pi | - | 3 |
|  |  |  | 10 | -36.6 | 1’ Pi |  |  |
|  |  |  | 10 | -38.5 | 1’ Pi |  |  |
|  |  |  | 10 | -36.9 | 6’ Pi |  |  |

| **Results of docking the potential activator ligands into the allosteric site of *Enterococcus faecalis* PYK** | | | | | | | | |
| --- | --- | --- | --- | --- | --- | --- | --- | --- |
| **Ligands** | **most favorable E_model_ score^*^**  **[kcal/mol]** | **Total number of binding poses** | **Probability of binding pose [%]^$^** | **E_model_  score per binding pose^§^ [kcal/mol]** | **Orientation of the P_i_-moiety^#^** | **Number of solutions**  **not clustered in a binding pose** | | **Number of solutions not docked in the allosteric site** |
| FBP | -81.4 | 5 | 30 | -76.8 | - | 3 | 2 | |
|  |  |  | 15 | -81.4 |  |  |  | |
|  |  |  | 5 | -79.5 |  |  |  | |
|  |  |  | 10 | -70.5 |  |  |  | |
|  |  |  | 15 | -71.3 |  |  |  | |
|  |  |  |  |  |  |  |  | |
| Gal6P | -67.9 | 4 | 25 | -67.9 | 6’ P_i_ | - | 1 | |
|  |  |  | 25 | -66.2 | 6’ P_i_ |  |  | |
|  |  |  | 15 | -60.6 | 6’ P_i_ |  |  | |
|  |  |  | 30 | -59.8 | 1’ P_i_ |  |  | |
|  |  |  |  |  |  |  |  | |
| G6P | -65.7 | 3 | 60 | -65.7 | 6’ P_i_ | - | 1 | |
|  |  |  | 15 | -54.7 | 6’ P_i_ |  |  | |
|  |  |  | 20 | -56.2 | 1’ P_i_ |  |  | |
|  |  |  |  |  |  |  |  | |
| F6P | -63.8 | 2 | 70 | -63.8 | 6’ P_i_ | - | 1 | |
|  |  |  | 25 | -55.4 | 1’ P_i_ |  |  | |
|  |  |  |  |  |  |  |  | |
| F1P | -62.4 | 4 | 25 | -62.4 | 6’ P_i_ | 2 | 4 | |
|  |  |  | 10 | -50.3 | 6’ P_i_ |  |  | |
|  |  |  | 10 | -56.5 | 1’ P_i_ |  |  | |
|  |  |  | 25 | -57.4 | 1’ P_i_ |  |  | |
|  |  |  |  |  |  |  |  | |
| R5P | -61.7 | 3 | 75 | -61.7 | 6’ P_i_ | - | 1 | |
|  |  |  | 15 | -50.5 | 6’ P_i_ |  |  | |
|  |  |  | 5 | -42.2 | 1’ P_i_ |  |  | |
|  |  |  |  |  |  |  |  | |
| Gal1P | -49.1 | 3 | 15 | -48.0 | 1’ P_i_ | - | 1 | |
|  |  |  | 15 | -47.3 | 1’ P_i_ |  |  | |
|  |  |  | 10 | -47.8 | 1’ P_i_ |  |  | |
