# Supplementary material for: Organism-Adapted Specificity of the Allosteric Regulation of Pyruvate Kinase in Lactic Acid Bacteria
Source: PLoS Comput Biol. 2013 Jul 25;9(7):e1003159. doi: 10.1371/journal.pcbi.1003159 (PMC3738050; doi:10.1371/journal.pcbi.1003159)
Supplement: Table S4 — Sequence information for the modelled PYKs. (DOCX) [file pcbi.1003159.s008.docx]

Supplementary Table S4:

| **Sequence information for the modeled PYKs** | | | | |
| --- | --- | --- | --- | --- |
| **Organism** | **Protein Entry Name** | **Uniprot Accession Number** | **EC number** | **Sequence Length**  [number of residues] |
| *Lactococcus lactis* | KPYK_LACLA | Q07637 | 2.7.1.40 | 502 |
| *Streptococcus mutans* | Q8DTX7_STRMU | Q8DTX7 | 2.7.1.40 | 500 |
| *Streptococcus pyogenes* | B5XLV5_STRPZ | B5XLV5 | 2.7.1.40 | 500 |
| *Enterococcus faecalis* | Q836R2_ENTFA | Q836R2 | 2.7.1.40 | 585 |
| *Lactobacillus plantarum* | Q88VY2_LACPL | Q88VY2 | 2.7.1.40 | 586 |
