# Supplementary material for: Organism-Adapted Specificity of the Allosteric Regulation of Pyruvate Kinase in Lactic Acid Bacteria
Source: PLoS Comput Biol. 2013 Jul 25;9(7):e1003159. doi: 10.1371/journal.pcbi.1003159 (PMC3738050; doi:10.1371/journal.pcbi.1003159)
Supplement: Table S5 — Assignment of residues from PYK crystal structures to the chimeric template for comparative modelling. (DOCX) [file pcbi.1003159.s009.docx]

Supplementary Table S5:

| **Assignment of residues from PYK crystal structures to the chimeric template for comparative modelling** | | |
| --- | --- | --- |
| chimeric template | *Bacillus stearothermophilus*  (2E28) | *Escherichia coli*  (1PKY) |
| 1 – 337 | 1 – 337 | - |
| 338 – 473 | - | 335 – 470 |
