# Supplementary material for: Organism-Adapted Specificity of the Allosteric Regulation of Pyruvate Kinase in Lactic Acid Bacteria
Source: PLoS Comput Biol. 2013 Jul 25;9(7):e1003159. doi: 10.1371/journal.pcbi.1003159 (PMC3738050; doi:10.1371/journal.pcbi.1003159)
Supplement: Table S6 — Residues excluded from comparative modelling of the PYKs. (DOCX) [file pcbi.1003159.s010.docx]

Supplementary Table S6:

| **Residues excluded from comparative modelling of the PYKs** | | | | |
| --- | --- | --- | --- | --- |
| **Organism** | **N-terminus** | **Loop** | **C-terminus** | **Extra C-terminal domain** |
| *Lactococcus lactis* | - | G12 – K40 | K502 | - |
| *Streptococcus mutans* | - | G12 – A40 | - | - |
| *Streptococcus pyogenes* | - | G12 – K40 | K500 | - |
| *Enterococcus faecalis* | M1 | - | - | G473 – I585 |
| *Lactobacillus plantarum* | M1 | - | - | G474 – L586 |
