# Supplementary material for: Organism-Adapted Specificity of the Allosteric Regulation of Pyruvate Kinase in Lactic Acid Bacteria
Source: PLoS Comput Biol. 2013 Jul 25;9(7):e1003159. doi: 10.1371/journal.pcbi.1003159 (PMC3738050; doi:10.1371/journal.pcbi.1003159)
Supplement: Table S7 — Flipped Residues and unusual protonation states introduced by WHATIF upon protonation of the PYK structures. (DOCX) [file pcbi.1003159.s011.docx]

Supplementary Table S7:

| **Flipped residues and unusual protonation states introduced by WHATIF upon protonation of the PYK structures** | | | | | | | | |
| --- | --- | --- | --- | --- | --- | --- | --- | --- |
|  | *Saccharomyces cerevisiae* PYK  (PDB id: 1A3W, A) | *Escherichia coli* PYK (PDB id: 1PKY, D) | Chimeric template | *Streptococcus mutans* PYK | *Lactococcus lactis* PYK | *Streptococcus pyogenes* PYK | *Lactobacillus plantarum* PYK | *Enterococcus faecalis* PYK |
| Flipped residues | N33 | Q45 |  |  |  |  |  |  |
|  | N46 | N155 | H41 | H33 | H33 | H62 | H42 | H42 |
|  | N46 | N195 | H44 | Q91 | Q118 | Q120 | Q76 | N48 |
|  | N97 | Q176 | N50 | Q118 | N188 | Q169 | H116 | N74 |
|  | N106 | H206 | H211 | N228 | N213 | H242 | Q147 | Q251 |
|  | H176 | N223 | H215 | Q335 | Q217 | Q244 | N187 | N228 |
|  | N180 | N228 | Q217 | N339 | N230 | N257 | H211 | N299 |
|  | N243 | Q260 | N230 | N348 | Q337 | N328 | Q261 | Q341 |
|  | Q244 | Q408 | Q411 | N353 | N384 | N382 | N229 | H382 |
|  | Q245 | N463 | N466 | N382 | N465 | N411 | N413 | Q438 |
|  | Q280 | H468 | H471 | N448 |  | N492 | N465 | N464 |
|  | N307 |  |  |  |  |  | Q470 | Q469 |
|  | N320 |  |  |  |  |  |  |  |
|  | Q357 |  |  |  |  |  |  |  |
|  | N364 |  |  |  |  |  |  |  |
|  | N493 |  |  |  |  |  |  |  |
| unusual protonation states | - | - | - | - | - | H147: double protonation | - | - |
