# Supplementary material for: Organism-Adapted Specificity of the Allosteric Regulation of Pyruvate Kinase in Lactic Acid Bacteria
Source: PLoS Comput Biol. 2013 Jul 25;9(7):e1003159. doi: 10.1371/journal.pcbi.1003159 (PMC3738050; doi:10.1371/journal.pcbi.1003159)
Supplement: Table S8 — Parameters of the kinetic model of the PYK from Lactococcus lactis . (DOCX) [file pcbi.1003159.s012.docx]

Supplementary Table S8:

| **Parameters of the kinetic model of the PYK from *Lactococcus lactis*** | | | |
| --- | --- | --- | --- |
| Parameter | Model parameter value | Experimentally determined value | Reference |
| *K_m_*(PEP) [mM] | 6.432 | 4 | [32] |
| *K_m_*(ADP) [mM] | 0.022 | 2.1 * | [10] |
| *K_m_*(PYR) [mM] | 0.593 | n.d. |  |
| *K_m_*(ATP) [mM] | 7.105 | n.d. |  |
| *V* [U/mg] | 0.403 | n.d. |  |
| *n* | 2.173 | 3.3 (PEP) 2.1 (ADP) | [32] |
| *k_a_*^$^ [mM] | 1.707 | (0.07- 0.2) | [10,32] |
| *k*_i_^$^ [mM] | 0.124 | (0.65 -1.3 ) | [10,32] |
| *β1* | 246.077 |  |  |
| *β2* | 0.0036 |  |  |
| *k_out_*^§^ [1/min] | 176381 |  |  |

*in the presence of 0.1 mM FBP

^$^ the model parameters describe the ratio of ligand binding and ligand dissociation and are therefore not directly comparable with experimentally measured k_a0.5v_ and k_i0.5v_ values.

^§^ represents the second reaction that consumes the product pyruvate (PYR) of the PYK reaction, following the experimental set-up; the reaction is described by mass action.
